# Reaction of 3-Amino-1,2,4-Triazole with Diethyl Phosphite and Triethyl Orthoformate: Acid-Base Properties and Antiosteoporotic Activities of the Products

**DOI:** 10.3390/molecules22020254

**Published:** 2017-02-08

**Authors:** Patrycja Miszczyk, Dorota Wieczorek, Joanna Gałęzowska, Błażej Dziuk, Joanna Wietrzyk, Ewa Chmielewska

**Affiliations:** 1 Department of Bioorganic Chemistry, Faculty of Chemistry, Wrocław University of Science and Technology, 50-370 Wrocław, Poland; patrycja.miszczyk@pwr.edu.pl; 2 Faculty of Chemistry, University of Opole, 45-040 Opole, Poland; Dorota.Wieczorek2@uni.opole.pl (D.W.); bdziuk@uni.opole.pl (B.D.); 3 Department of Inorganic Chemistry, Faculty of Pharmacy, Wrocław Medical University, 50-556 Wrocław, Poland; joanna.galezowska@umed.wroc.pl; 4 Ludwik Hirszfeld Institute of Immunology and Experimental Therapy, Polish Academy of Sciences, 53-114 Wrocław, Poland; wietrzyk@iitd.pan.wroc.pl

**Keywords:** organophosphorus chemistry, bisphosphonic acids, aminomethylenebisphosponates, three-component reaction, triazole, P-containing drugs, anti-proliferative activity, osteoclasts, UV-Vis spectroscopy, pH-titration, potentiometry, crystallography

## Abstract

The reaction of diethyl phosphite with triethyl orthoformate and a primary amine followed by hydrolysis is presented, and the reaction was suitable for the preparation of (aminomethylene)bisphosphonates. 3-Amino-1,2,4-triazole was chosen as an interesting substrate for this reaction because it possesses multiple groups that can serve as the amino component in the reaction—namely, the side-chain and triazole amines. This substrate readily forms 1,2,4-triazolyl-3-yl-aminomethylenebisphosphonic acid (compound **1**) as a major product, along with *N*-ethylated bisphosphonates as side products. The in vitro antiproliferative effects of the synthesized aminomethylenebisphosphonic acids against J774E macrophages were determined. These compounds exhibit similar activity to zoledronic acid and higher activity than incadronic acid.

## 1. Introduction

Bisphosphonic acids constitute a class of compounds that exhibit a wide variety of biological activities. The most studied of these compounds are those related to bone-formation disorders. These studies have resulted in the discovery of numerous commercially available drugs that prevent bone mass loss and are used against osteoporosis, Paget’s disease, multiple myeloma and other conditions involving fragile, breakable bones [1,2,3]. Aminomethylenebisphosphonates are a subclass of bisphosphonates, and representatives of this group exhibit promising and variable physiological activities, such that they may be used as antibacterials, anticancer agents, pain palliation drugs and herbicides [1].

Despite low or average yields, harsh conditions and problems for the separation of pure esters, a simple three-component condensation of stoichiometric amounts of an amine, diethyl phosphite and triethyl orthoformate is the most common procedure for the preparation of a wide variety of aminomethylenebisphosphonic acids [4,5,6,7]. There are several other less useful methods, such as the reaction of dialkylphosphonates with acetals and *N*-substituted formamides [8,9], the phosphorylation of the product of the Beckman rearrangement of oxime [10] or the reaction of trialkylphosphites with chloroiminium salts [11,12,13,14]. Because the three-component reaction usually yields a complex mixture of products that are difficult to separate, the resulting esters are not isolated. Instead, the crude reaction mixture is hydrolysed, yielding the bisphosphonic acid, which is subsequently isolated. This reaction is also quite unpredictable and frequently affords unexpected products, the composition of which depends on the applied conditions (molar ratio of substrates, temperature and reaction time) [7]. This is also observed when 3-amino-1,2,4-triazole is used as a substrate.

Because of the difficulty in isolating and culturing large numbers of osteoclasts, many studies that characterize the pharmacological properties of bisphosphonates in vitro are performed in osteoclast surrogates, e.g., macrophages with J774E. These macrophages and osteoclasts are both derived from the haematopoietic lineage and are highly endocytic and capable of demineralizing bone particles [15].

## 2. Results and Discussion

### 2.1. Chemistry

3-Amino-1,2,4-triazole is an interesting substrate for the reaction with triethyl orthoformate and diethyl phosphite because it possesses multiple groups that can serve as the amino component in the reaction—namely, the amino moiety in the side-chain and the triazole amine. Moreover, due to tautomerism [16,17,18,19,20], three positions of the triazole ring can react.

**Figure 1:**
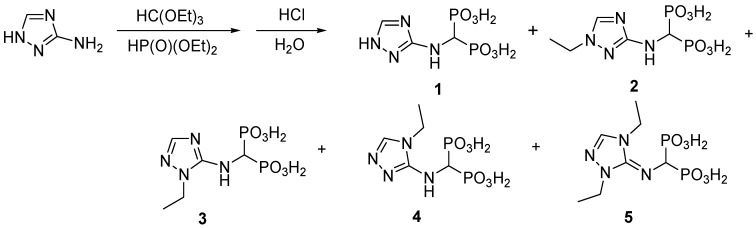
Reaction of 3-amino-1,2,4-triazole with tetraethyl orthoformate and diethyl phosphite.

If two amino groups of the substrate are considered reactive, then the required stoichiometric amount of the amine to phosphite to orthoformate is 1:4:2. NMR analysis of the crude reaction mixture, obtained after hydrolysis, indicated the formation of the expected mono-substituted 1,2,4-triazolyl-3-yl-aminomethylenebisphosphonic acid (compound **1**) as the major product and a mixture of its *N*-ethylated compounds (likely **2**, **3**, **4** and **5**; Figure 1). The *N*-ethylated products are easily observed in the ^1^H-NMR spectra of the crude reaction mixture as well-separated ethyl groups (see Supplementary Materials). Their formation was additionally confirmed by mass spectrometry (see Supplementary Materials). The *N*-ethylation of bisphosphonate 1 was not surprising, considering that diethyl phosphite is known to act as an alkylating agent [6,7].

**Figure 2:**
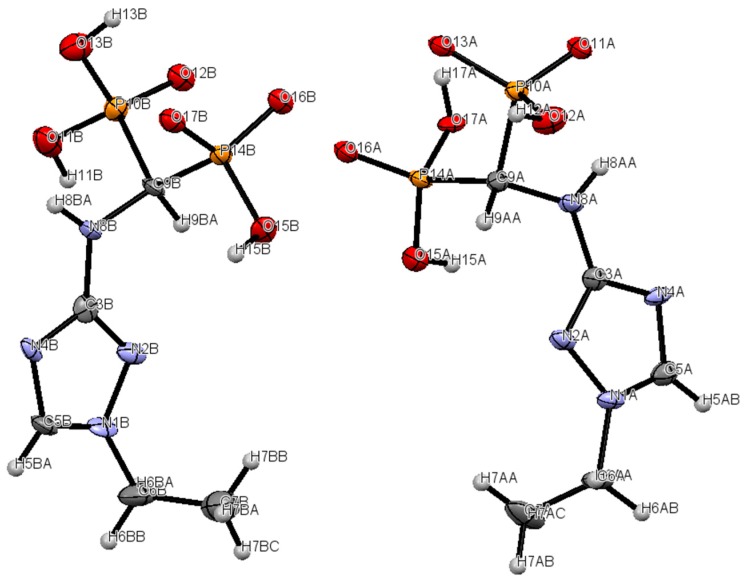
Compound **2** in the asymmetric part of unit cell. Thermal ellipsoids are shown at the 50% probability level.

**Figure 3:**
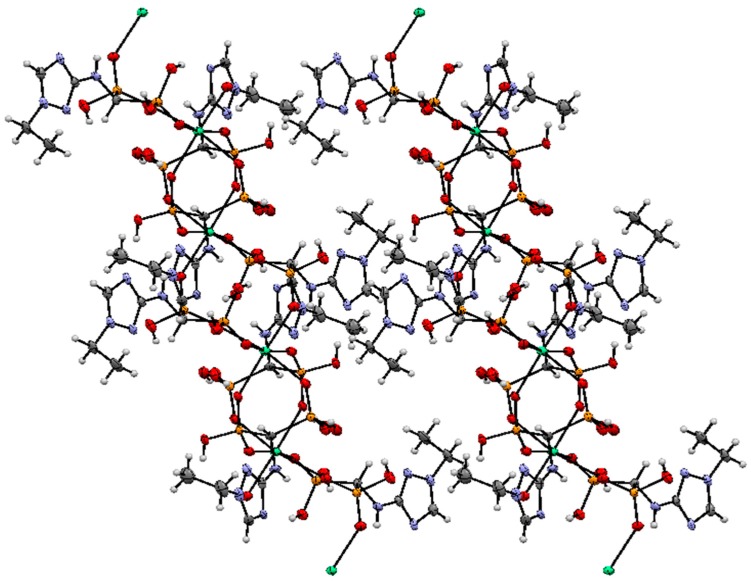
The crystal packing of compound **2** viewed along the *b* axis.

We succeeded in isolating of one of these compounds (compound **2**). Its structure was determined by X-ray analysis (Figure 2 and Figure 3). Interestingly, the disappearance of the signal related to the proton at position 5 in compound **2** after prolonged (several days) storage in D_2_O/NaOD solution was also observed (see Supplementary Materials). This was due to the exchange of this proton with deuterium.

For this reaction, the influence of reaction conditions was studied in some detail, and the obtained results are collected in Table 1. As seen from Table 1, in all reaction conditions, compound **1** was produced as the predominate product with the formation of the *N*-ethylated products dependent on the ratio of the substrates.

### 2.2. Crystallography

Crystallographic data for compound **2** is given in Table 1. The solid-state structure of calcium(II) 1,2,4-triazolyl3-yl-(*N*-ethyl)aminomethylenebisphosphonate (**2**) was determined by X-ray crystallography. The molecular structure of the 1,2,4-triazolyl-3-yl-(*N*-ethyl)-aminomethylenebisphosphonate ligand and the atomic numbering scheme are presented in Figure 2, and the crystal packing is shown in Figure 3. In the unit cell, there are two crystallographically independent ligands which are octahedrally coordinated by a Ca^2+^ ion. The structure of the octahedron is distorted. Compound **2** complexed calcium ions from the water used for crystallization. Additionally, in the structure, there are four disordered water molecules, which are located in the channels formed between the molecules of the unit cell.

### 2.3. Potentiometry

The acid-base properties of compounds **1** and **2** were determined to understand their behaviour in aqueous solutions. These studies were performed using potentiometry prompted by pH-UV titration studies and, for compound **1**, pH*-NMR titration studies (pH* = pH uncorrected for the isotopic effect). Calculated protonation values (p*K*s) are given in Table 2.

**Figure 4:**
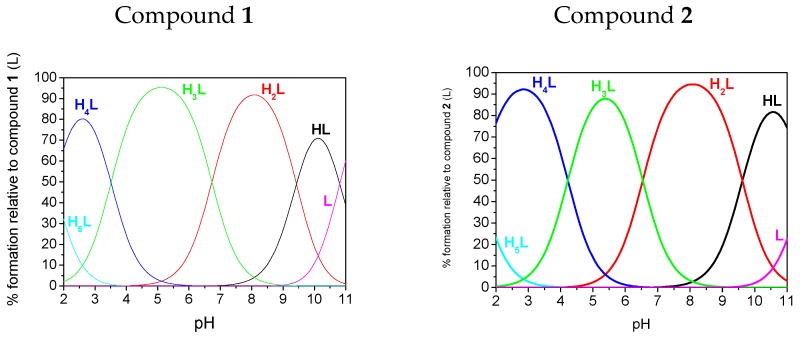
Species distribution diagrams of the ligands calculated based on potentiometric results.

The fully protonated forms of both compounds possess six dissociable protons (H_6_L): four on the bisphosphonic functional group, one on the 1,2,4-triazole ring and one located on the central tertiary N amine atom. However, we were only able to determine five protonation constants (p*K*_1_–p*K*_5_) because p*K*_6_, which corresponds to the dissociation of one proton from the PO_3_H_2_ group, is strongly acidic and could not be determined under the conditions of our pH measurements (pH 2–11). Species distribution diagrams are depicted in Figure 4. For the case of both ligands, three protonation steps of phosphonic groups were found from the p*K*s values, which agree well with previous studies on *N*-(pyridinyl)bisphosphonates [21,22] that are only slightly lower in value. Based on the comparison of the preliminary assignments, which assume that the highest estimated p*K*_1_ value for compound **1** belongs to the amine, the next three (p*K*_2_ = 9.62, p*K*_3_ = 6.54 and p*K*_5_ = 1.48) values correspond to the bisphosphonic functional group, and the remaining value (p*K*_4_ = 4.22) belongs to the 1,2,4-triazole ring. A significant reduction of the p*K* value of the triazole functional group (p*K*_4_ values of 4.52 and 3.51, respectively, for compounds **1** and **2**) in comparison to the unsubstituted triazole (p*K* = 9.26 for 1,2,3-triazole [23] and p*K* = 9.95 [24] for 1,2,4-triazole) was also observed previously for substituted triazoles. The previously reported values were 4.20 for 3-amino-1,2,4-triazole [19,24] and approximately 3 for the whole family of 1,2,3-triazole-pyridines (for example, p*K* = 3.40 was found for 3-(4-(pyridine-2-yl)-1*H*-1,2,3-triazol-1-yl-propan-1-ol) [25]. Species distribution diagrams (Figure 4) indicate that the monoprotonated phosphonic groups (forms H_3_L and H_2_L) dominate in a broad range of pH values.

The results of the potentiometric studies are supported by spectroscopic determination of the p*K* values of the studied compounds. The results, obtained from electronic spectra generated in the HypSpec program for the detected species [26], are presented in Table 2 and Figure S1. Unsubstituted 1,2,4-triazole shows a very weak absorption at 205 nm in the ultraviolet absorption spectrum, which shifts bathochromically with triazole substitution, e.g., to 221 nm for *N*-acetyl-1,2,4-triazole [27]. For compound **1**, the band at approximately 215 nm was already present under a very acidic pH value (pH 1.57) (Figure 4). This band is assigned to the protonated 1,2,4-triazole. The band underwent a bathochromic shift to 230 nm when the pH rises, which is assigned to the H_4_L → H_3_L^−^ + H^+^ deprotonation process at a p*K*_4_ of 4.22 or 4.16 (estimated potentiometrically or spectrophotometrically, respectively). This was the biggest change in absorption for the spectroscopic determination by UV spectrophotometry. This change was the reason for assigning the p*K*_4_ value to the deprotonation of 1,2,4-triazole. Adequate changes occur for the spectra of compound **2**. The triazole band was located at approximately 230 nm (represented by the H_3_L species) and underwent a bathochromic shift to 245 nm at slightly lower pH than was observed for compound **1** (p*K*_4_ = 3.51 or 3.32 by potentiometric or spectrophotometric estimation, respectively). Generally, spectroscopic titrations (see Figure S23 Supplementary Materials) are in a good agreement with potentiometric titrations and provide similar p*K* values.

To comprehend the solution behaviour of the compounds, NMR spectra of compound **1** were monitored over a broad range of pH* (the results are presented in Figure S24, Supplementary Materials). The studied compounds can be considered a family, in which, for both compounds, each of the phosphonate groups can accept two protons, and the heterocyclic nitrogen atom can accept one proton. The goal of these experiments was not to determine all protonation constants but only to confirm the protonation scheme. The shifts of the ^31^P phosphorus nuclei reflected the protonation process for almost all groups (Figure S24A) and revealed the protonation processes at a pH below 2, in the pH range of 4–6 and in the pH range of 10–12. For the ^1^H nuclei, we followed the shifts on the aromatic ring of compound **1** (Figure S24B). The biggest change fell in the pH range of 4–5, confirming the acidic deprotonation of the triazole ring. Although the concentration of the studied compound was far higher in the NMR studies than in the potentiometric studies, which caused precipitation to occur above pH 10, the obtained p*K* values are in good agreement with those obtained by potentiometry.

### 2.4. In Vitro Evaluation

To screen for potential antiosteoporotic activity of the bisphosphonates, their antiproliferative activity towards in vitro cell cultures was determined. J774E macrophages and osteoclasts are both derived from haematopoietic lineage and are highly endocytic and capable of demineralizing bone particles [15]. Therefore, they are models for studies on the influence of bisphosphonates on the proliferation and activity of tumour-associated macrophages (TAMs) [28]. For this purpose, mouse macrophage-like J774E cells, originating from the same precursors as the osteoclasts, were used [29,30]. Such cells are well recognized for being sensitive to bisphosphonates, which likely act by inducing apoptosis in the cells. The J744E cell line was obtained from a cell bank at the Ludwik Hirszfeld Institute of Immunology and Experimental Therapy, Polish Academy of Sciences.

Both compounds were moderately active, with IC_50_ values of 24.88 ± 5.17 (for compound **1**) and 30.58 ± 4.64 (for compound **2**) μM. Comparing the activities of compounds **1** and **2** with the activities of control samples, zoledronic acid (24.51 ± 2.23 μM) and incadronic acid (47.85 ± 1.39 μM), indicates that compounds **1** and **2** are promising antiosteoporotic drug candidates.

## 3. Experimental Section

### 3.1. General Information

All solvents and reagents were purchased from commercial suppliers, were of analytical grade and were used without further purification. Unless otherwise specified, solvents were removed with a rotary evaporator. The ^1^H-, ^31^P- and ^13^C-NMR spectroscopic experiments were performed on a Bruker Ultrashield Spectrometer (Bruker, Rheinstetten, Germany) operating at 400.13 (^1^H) MHz, 161.98 MHz (^31^P{^1^H}) and 151.016 MHz (^13^C) or a Bruker Avance II Ultrashield Plus (Bruker, Rheinstetten, Germany) 600.58 MHz (^1^H), 243.12 MHz (^31^P{^1^H}) and 100.61 MHz (^13^C). Measurements were made in D_2_O with NaOD (99.9 at. %D) solutions at 300 K, and all solvents were supplied by ARMAR AG (Dottingen, Switzerland). Chemical shifts are reported in ppm relative to TMS and 85% H_3_PO_4_, used as external standards, while coupling constants are reported in Hz. Melting points were determined on an SRS Melting Point Apparatus OptiMelt MPA 100 (Stanford Research Systems, Sunnyvale, CA, USA) and are reported without correction. Mass spectra were recorded at the Faculty of Chemistry, Wroclaw University of Science and Technology using a Waters LCT Premier XE mass spectrometer (method of ionization ESI, (Waters, Milford, MA, USA). Infrared spectra were recorded on an FT-IR Bruker Vertex 70/70 V Spectrometer (Bruker, Ettlingen, Germany).

### 3.2. Syntheses

#### 3.2.1. Synthesis of 1,2,4-Triazolyl-3-yl-Aminomethylenebisphosphonic Acid (Compound **1**)

3-Amino-1,2,4 triazole (0.03 mol, 2.52 g), triethyl orthoformate (0.12 mol, 8.80 mL) and diethyl phosphite (0.18 mol, 23.31 mL) were heated and simultaneously stirred at a temperature of ~130 °C on a heating plate (125 °C in the reaction medium) of a Radley’s Carousel apparatus overnight (15 h). The mixture was cooled, and the volatile components were removed using a rotary evaporator. The resulting mixture was dissolved in ethyl acetate (100 mL) and purified by washing with water (100 mL), saturated sodium chloride solution (100 mL) and again with water (100 mL). The solution was dried over anhydrous MgSO_4_, and the solvent was evaporated under vacuum. Boiling in 20 mL of 6 N hydrochloric acid for 12 h hydrolysed the resulting crude reaction product. After cooling, the volatile components were removed using a rotary evaporator, and the resulting oil was dissolved in a minimal amount of hot water, decoloured with activated charcoal and purified by crystallization from a water/ethanol mixture (80/20 *v*/*v*).

Compound **1** was obtained as a white solid; yield: 37%; m.p. 291–292 °C; ^31^P-NMR (161.98 MHz, NaOD, ppm): δ = 14.92; ^1^H-NMR (400.13 MHz, NaOD, ppm): δ = 3.83 (t, 1H, *J* = 19.44, C**H**P_2_), 7.58 (s, 1H, C**H**N), ^13^C-NMR (151.02 MHz, NaOD, ppm): δ = 52.27 (t, *J* = 127.91 Hz, **C**HP_2_), 146.68, 157.92, HRMS (TOF MS ESI^−^): [M − H]^−^ Calcd. for C_3_H_8_N_4_O_6_P_2_: 256.9841, found: 256.9853. IR (cm^−1^): 3600–2400 (broad peak, hydrogen bonds), 1674 (NH), 1195 (P=O), 1146 (P=O), 947 and 914 (POH-).

#### 3.2.2. Synthesis of 1,2,4-Triazolyl-3-yl-(*N*-Ethyl)aminomethylenebisphosphonic Acid (**2**)

3-Amino-1,2,4 triazole (0.03 mol, 2.52 g), triethyl orthoformate (0.06 mol, 9.63 mL) and diethyl phosphite (0.12 mol, 15.50 mL) were heated and simultaneously stirred at a temperature of ~130 °C on a heating plate (125 °C in the reaction medium) of a Radley’s Carousel apparatus overnight (15 h, Radleys, Essex, UK). The mixture was cooled, and the volatile components were removed using a rotary evaporator. The resulting mixture was dissolved in ethyl acetate (100 mL) and purified by washing with water (100 mL), saturated sodium chloride solution (100 mL) and again with water (100 mL). The solution was dried over anhydrous MgSO_4_, and the solvent was evaporated under vacuum. Boiling in 20 mL of 6 N hydrochloric acid for 12 h hydrolysed the resulting crude reaction product. After cooling, the volatile components were removed using a rotary evaporator, and the resulting oil was dissolved in minimal amount of water (30 mL), decoloured with activated charcoal and purified by crystallization from hot water. The impure product was mixed with water for 5 days until the dissolution of impurities was observed and then was filtered and washed with distilled water and dried in vacuo.

Compound **2** was obtained as a creamy solid; yield: 21%; m.p. 297–298 °C; ^31^P-NMR (243.12 MHz, D_2_O +NaOD, ppm): δ = 15.82; ^1^H-NMR (600.58 MHz, D_2_O + NaOD, ppm): δ = 1.31 (t, 3H, *J* = 7.28 Hz, C**H**_3_), 3.59 (t, 1H, *J* = 19.62, C**H**P_2_), 3.92 (q, 2H, *J* = 7.26 Hz, C**H**_2_), 7.82 (s, 1H, C**H**N); ^13^C-NMR (151.02 MHz, D_2_O + NaOD, ppm): δ = 13.87, 44.18, 53.99 (t, *J* = 123.98 Hz, **C**HP_2_), 142.27, 164.73; HRMS (TOF MS ESI^−^): [M − H]^−^ Calcd. for C_5_H_12_N_4_O_6_P_2_: 287.0310, found: 287.0316. IR (cm^−1^): 3450–2500 (broad, hydrogen bonds), 3383 (NH), 3103 (NH), 1631 (NH), 1587 (NH), 1221(P + O), 1142 (POH-).

### 3.3. Antiproliferative Activity

This experiment was performed according to a previously published procedure [31]. To use the cell line, it is necessary to first thaw the cells using a water bath at 37 °C and gentle stirring. The cells were placed into the culture medium. This line was passaged 2 times a week in RPMI (growth medium consisting of 10% FBS, 100 μg/mL streptomycin, 100 U/mL penicillin and 2 mM glutamine) in Petri dishes. The cell growth was monitored under a microscope, and part of the material was used in the next stage of the experiment. Then, the cells were separated from the bottom (the surface proteins present on the plasma membrane allowing adhesion were destroyed) and were collected from the culture vessel. Then, in a Bürker chamber, the cell density was counted using a drop of cell suspension in a mixture ratio of 1:1 with trypan blue.

Then, a 96-well microtiter plate assay was performed with 100 μL of cells (5 × 10^4^/mL) in a cultivation solution followed, after 24 h, by the addition of 100 μL of a solution of the tested compound (10^−3^, 10^−4^, 10^−5^, or 10^−6^ M) in the same medium. Plates were incubated at 37 °C in a humidified atmosphere saturated with 5% CO_2_. The in vitro cytotoxicities of all agents were examined by colorimetric MTT assay after 72 h of exposure of the cultured cells to varying concentrations of the tested compounds [32]. Briefly, 20 μL of the MTT solution (MTT: 3-(4,5-dimethylthiazol-2-yl)-2,5-diphenyl tetrazolium bromide) was added to each well and incubated for 4 h. After the incubation period was completed, 80 μL of the lysing mixture was added to each well (lysing mixture: 225 mL dimethylformamide, 67.5 g sodium dodecyl sulfate and 275 mL of distilled water). The optical densities of the samples were determined after 24 h using a Multiskan RC photometer (Labsystems, Helsinki, Finland) at 570 nm.

### 3.4. NMR Measurements

Samples for ^1^H and ^31^P{^1^H} NMR titration studies were prepared in D_2_O. Titrations were performed over a pH range of ca. 2–13. The pH was measured using a pH meter (Model PHS-3E M83, Shanghai San-Xin Instrumentation, Shanghai, China) equipped with a Hydromet ERH-13–6 combined electrode (Gliwice, Poland), and the pH was given in a meter reading without correction for pD.

### 3.5. UV–Vis Measurements

Absorption spectra for the compounds were collected using a Varian Cary 50 spectrophotometer (Varian, Mulgrave, Victoria, Australia) in Quartz Hellma curvets with a 1 cm optical path. The solutions were prepared by dissolving the appropriate concentration of ligands ([L] = 7 × 10^−5^ mol·dm^−3^) in 0.004 M HCl with 0.1 M ionic strength (KCl). The background electrolyte was used as a baseline reference. Solution pH was changed manually by adding small amounts of concentrated potassium hydroxide and checked the pH using the 913 Metrohm pH meter equipped with a combined glass electrode (Metrohm 6.0224.100, Herisau, Switzerland). The protonation constants and spectral deconvolution were refined using the least squares fitting program HypSpec [26]. Factor analysis by the HypSpec software (http://www.hyperquad.co.uk/) was implemented to characterize the number of species present in the solution.

### 3.6. Potentiometric Studies

The titration studies of the compounds were carried out in double-distilled water using a Metrohm 809 Titrando system equipped with a combined glass electrode (Metrohm 6.0224.100) which was calibrated daily for hydrogen concentration using HCl (Merck, Warsow, Poland) (0.004 M) according to the procedure of Irving et al. [33]. The ionic strength was fixed at *I* = 0.1 M with KCl (POCh). Alkali, CO_2_-free 0.1017 M KOH solution (POCh) was standardized by titration with potassium hydrogen phthalate (Merck). The ionic product of water under the experimental conditions was 10^−13.77^ mol^2^·dm^−6^. The purity and exact concentration of each ligand were determined by the method of Gran [34,35]. The titrations were carried out on 2.0 mL samples in a thermostatted cell at 25 ± 0.2 °C under a stream of Ar. The HYPERQUAD2013 computer program [36] was applied to calculate the protonation constants. Triplicate titrations of each ligand (177–222 points) in a concentration of 1.0 × 10^−3^ mol·dm^−3^ were performed and used for the calculations. The distribution curves of the protonated species of the compounds (marked as L) as a function of pH were calculated using the HySS2009 program [37].

### 3.7. Crystallography

Relevant crystallographic data for the molecules and the full geometrical information are summarized in Table 3, Table S1 and Table S2 of the Supplementary Materials. The single-crystal X-ray diffraction experiments were performed at 100.0(1) K on an Xcalibur diffractometer (Rigaku Oxford Diffraction, Sevenoaks, Kent, UK), equipped with a CCD detector and a graphite monochromator (Rigaku Oxford Diffraction) with Mo Kα radiation and furnished with an Oxford Cryosystem N2 gas stream device. The reciprocal space was explored by ω scans. The reflections were measured with a radiation exposure time from 4 to 25 s, according to diffraction intensities. The detector was positioned at a 60-mm distance from the crystal. Procession of the diffraction data was performed using the CrysAlis CCD [38]. The structure of Cu(II) 1,2,4-triazolyl-3-yl-(*N*-ethyl)aminomethylenebisphosphonate was solved in the P-1 space group by direct methods and refined by a full-matrix least-squares method using the SHELXL14 program [39]. Lorentz and polarization corrections were applied. Non-hydrogen atoms were refined anisotropically. In structures, H atoms were refined using a riding model. The structure drawings were prepared using the SHELXTL and Mercury programs [40].

The crystallographic data for Cu(II) 1,2,4-triazolyl-3-yl-(*N*-ethyl)aminomethylenebisphosphonate have been deposited at the Cambridge Crystallographic Data Centre as supplementary publication no. CCDC 1516180. These data can be obtained free of charge via http://www.ccdc.cam.ac.uk/conts/retrieving.html, or from the Cambridge Crystallographic Data Centre, 12 Union Road, Cambridge CB2 1EZ, UK; fax: 144 1223 336 033; email:deposit@ccdc.cam.ac.uk.

## 4. Conclusions

The reaction of 3-amino-1,2,4-triazole with triethyl orthoformate and diethyl phosphite was straightforward and resulted in the formation of the expected 1,2,4-triazoly-3-yl-aminomethylenebisphosphonic acid (compound **1**); however, the reaction was accompanied by the production of significant quantities of *N*-ethylated products (confirmed by isolation). Acid-base properties of both isolated compound **1** and **2** were determined using potentiometry and UV and NMR titrations.

Despite the potential for substitution of the ethyl functional group in the 1,2,4-triazole ring, it was determined that compound **2**, as well as compound **1**, possessed an acidic proton on the triazole ring, which is available for H-bonding.

Both ligands released protons in a wide range of pH values (p*K* values vary between ~1 up to ~11), which can promote the creation of intermolecular H-bonds. In pH 7.4, which is of biological relevance, both studied compounds are doubly protonated with the protons located on the phosphonic moiety only.

The obtained aminomethylenebisphosphonic acids revealed interesting activity, especially compound **1**, which is of particular interest for anti-osteolytic therapy, as powerful inhibitors of the activity of J774E cells (IC_50_ value 25 μM for compound **1**). Thus, compound **1** is equipotent to the popular drug zoledronate and exhibits higher activity than the drug incadronate.

## Figures and Tables

**Table 1:** Yields and ratios of the products of the reaction between 3-amino-1,2,4-triazole, triethyl orthoformate and diethyl phosphite.

Ratio of Substrates *Amine/triethyl orthoformate/diethyl phosphate*	1:1:2	1:2:4	1:4:6
Temperature of Reaction (°C)	130	130	130
Time of Reaction (h)	15	15	15
Ratio of Products 1: (2 + 3 + 4 + 5) Determined from ^1^H-NMR Spectra by Integration of Triazole Protons	1:0.7	1:0.3	1:0.1
Yield of Isolated Compounds (%)	27% (compound **1**)	21% (compound **2**)	37% (compound **1**)

**Table 2:** The protonation constants of **1** and **2** measured in 0.1 M KCl at 25 °C. Concentrations of the compounds were as following: [L]_potentiometry_ = 1 × 10^−3^ M, [L]_UV-Vis_ = 7 × 10^−5^ M.

Assignment	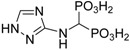		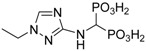	
	1		2	
	Potentiometry	UV	Potentiometry	UV
p*K*_1_(HL)_amine_	11.52(3)	11.68(2)	10.81(1)	nd
p*K*_2_(H_2_L)_phosphonate_	9.62(3)	9.32(5)	9.43(1)	9.56(2)
p*K*_3_(H_3_L)_phosphonate_	6.54(4)	6.15(6)	6.73(1)	5.90(2)
p*K*_4_(H_4_L)_triazole_	4.22(4)	4.16(6)	3.51(1)	3.32(2)
p*K*_5_(H_5_L)_phosphonate_	1.48(4)	0.67(6)	1.69(1)	nd

The reported errors on p*K* values are given as 1σ.

**Table 3:** X-ray crystallography experimental details.

Crystal Data	
Chemical formula	C_10_H_30_CaN_8_O_16_P_4_
M_r_	682.38
Crystal system, space group	Triclinic, P-1
Temperature (K)	100.0(1)
*a*, *b*, *c* (Å)	10.6815 (4), 12.5015 (6), 12.6155 (5)
α, β, γ (°)	60.518 (5), 66.986 (4), 69.072 (4)
*V* (Å^3^)	1320.90 (12)
*Z*	2
Radiation type	Mo *K*α
μ (mm^−1^)	0.57
Crystal size (mm)	0.02 × 0.01 × 0.01
Data collection	
Diffractometer	Oxford Xcalibur
No. of measured, independent and observed [I > 2σ(I)] reflections	8995, 5080, 2769
*R*_int_	0.079
(sin θ*/*λ)_max_ (Å^−1^)	0.617
Refinement	
*R*[*F*^2^ > *2*σ*(F*^2^*)*], *wR*(*F*^2^), *S*	0.0790, 0.1307, 1.031
No. of reflections	5080
No. of parameters	420
Δρ_max_, Δρ_min_ (e Å^−3^)	0.66, −0.77

## Supplementary Materials

Supplementary materials can be accessed at: http://www.mdpi.com/1420-3049/22/2/254/s1.
