# Supplementary material for: Reaction of 3-Amino-1,2,4-Triazole with Diethyl Phosphite and Triethyl Orthoformate: Acid-Base Properties and Antiosteoporotic Activities of the Products
Source: Molecules. 2017 Feb 8;22(2):254. doi: 10.3390/molecules22020254 (PMC6155913; doi:10.3390/molecules22020254)
Supplement: Supplementary file 1 [file molecules-22-00254-s001.pdf]

Supplementary Materials: Reaction of 3-Amino-1,2,4-Triazole with Diethyl Phosphite and Triethyl Orthoformate: Acid-Base Properties and Antiosteoporotic Activities of the Products

Patrycja Miszczyk, Dorota Wieczorek, Joanna Gałęzowska, Błażej Dziuk, Joanna Wietrzyk and Ewa Chmielewska

1. Spectroscopic Data

Compound **1**

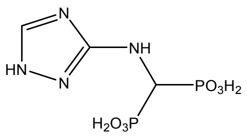

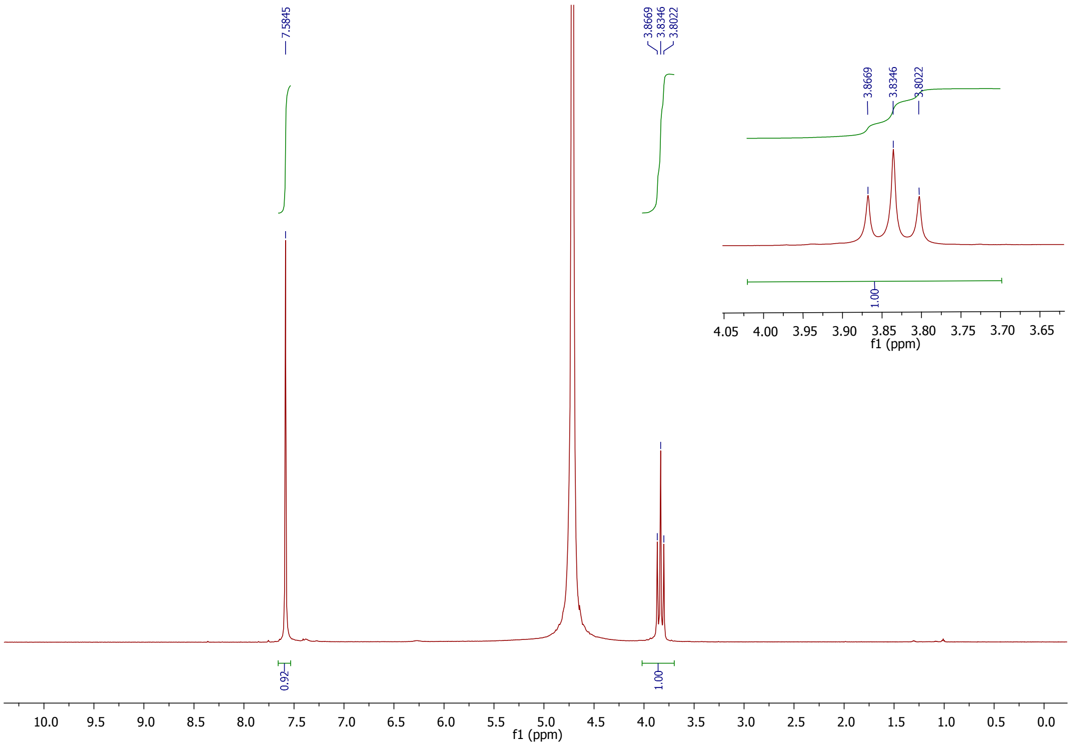

Figure S1. ^{1}H-NMR spectrum of compound **1**.

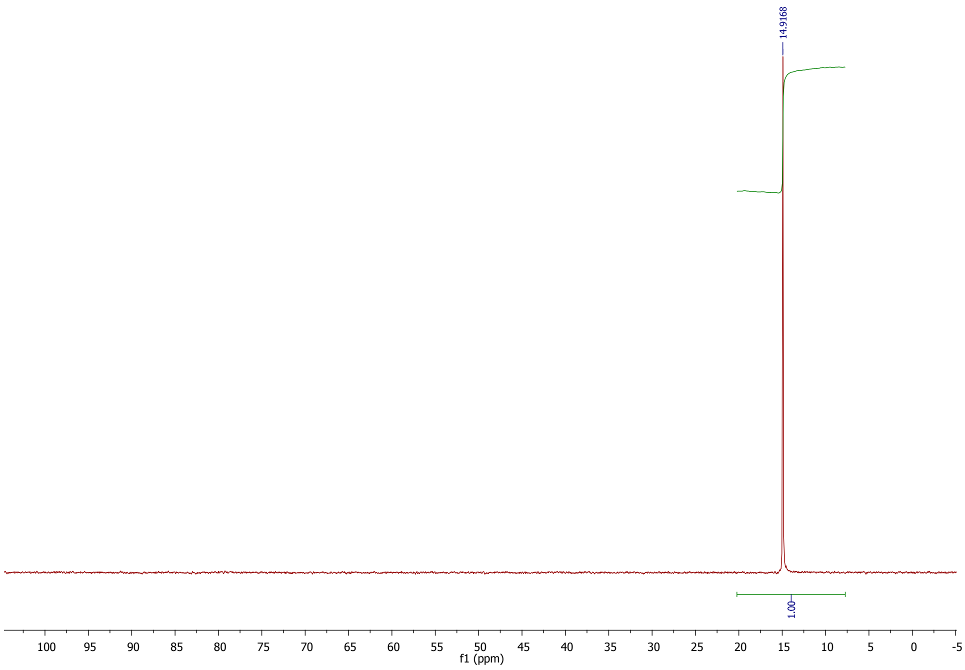

Figure S2. ^{31}P-NMR spectrum of compound **1.**

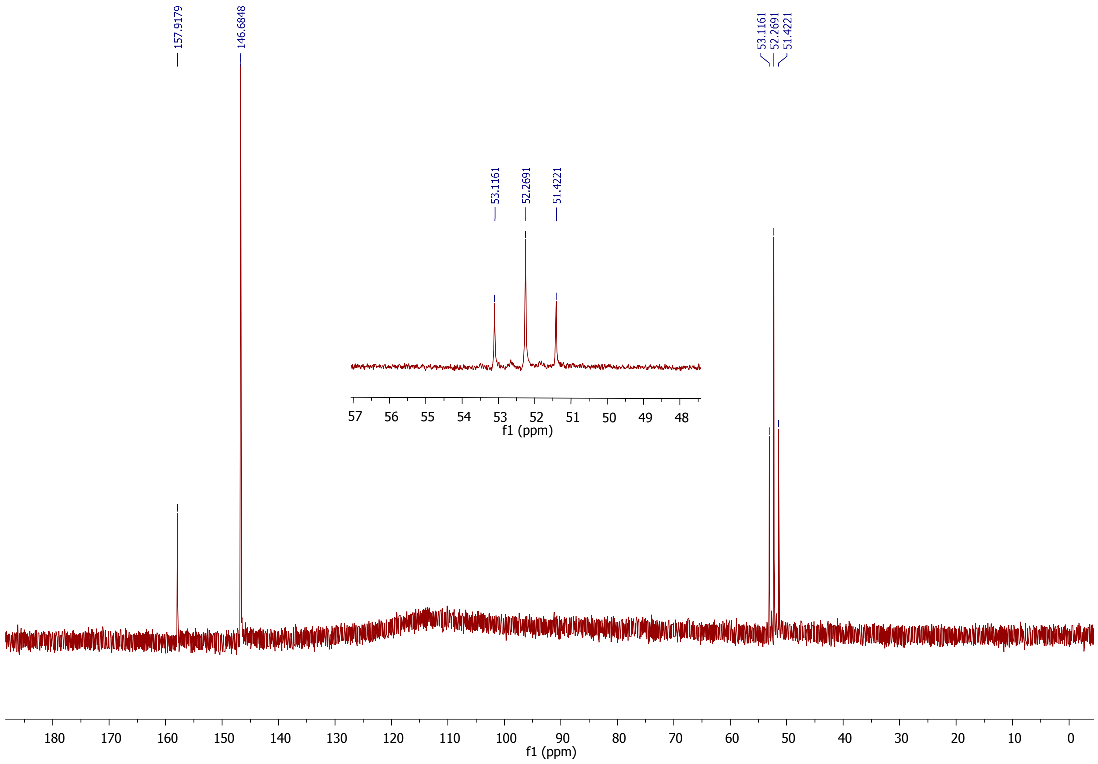

Figure S3. ^{13}C-NMR spectrum of compound **1**.

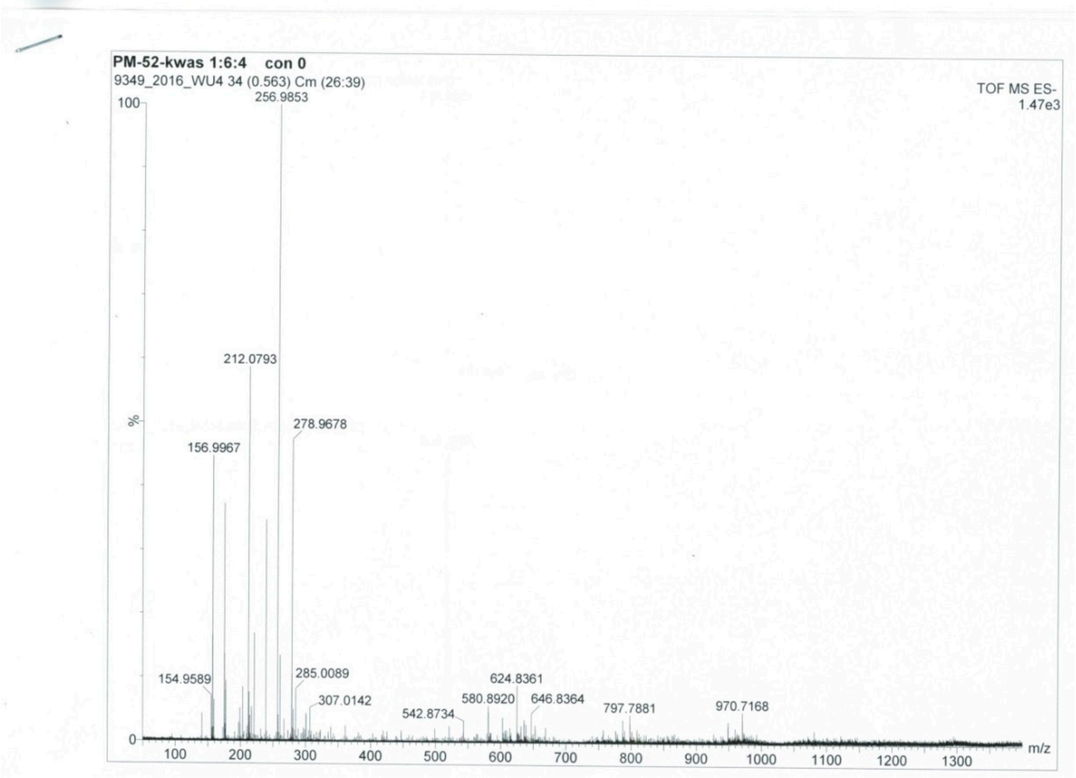

Figure S4. HRMS spectrum of compound **1.**

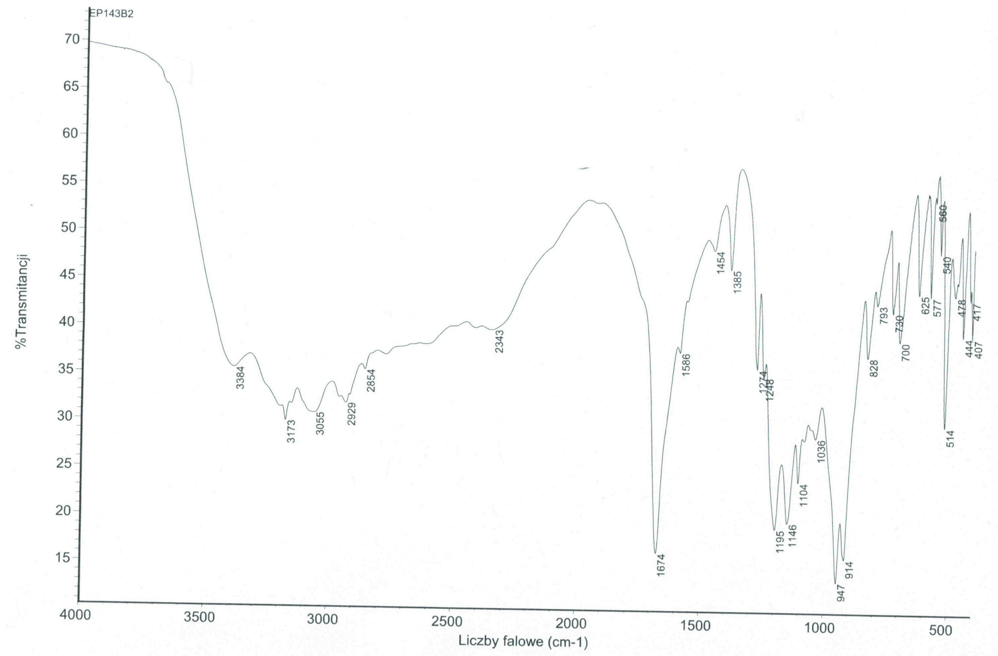

Figure S5. IR spectrum of compound **1.**

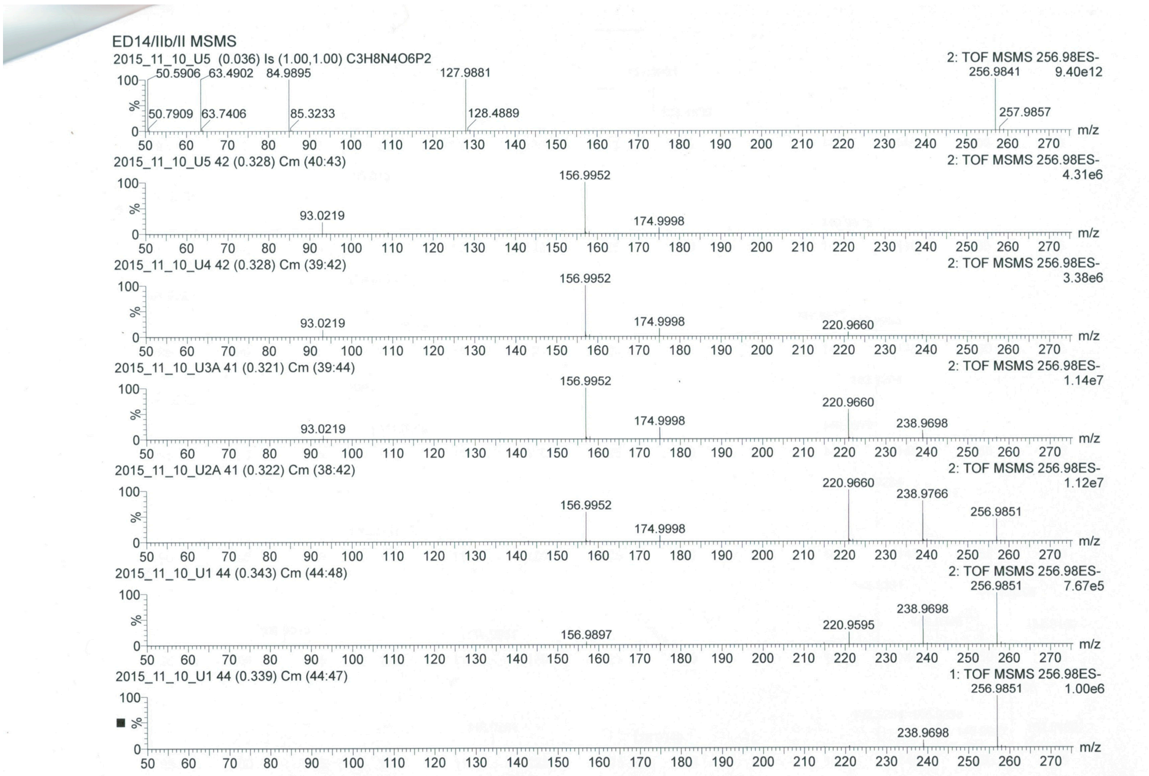

Figure S6. MS spectrum with fragmentation, for compound **1** (method of ionization (−)).

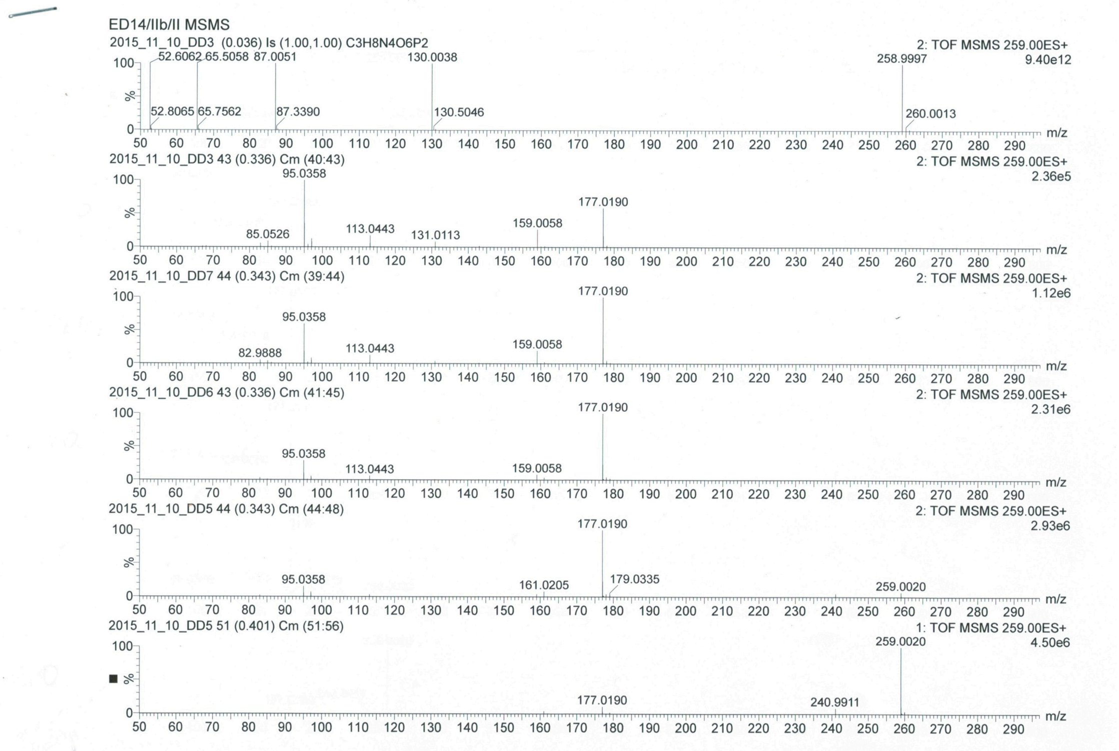

Figure S7. MS spectrum with fragmentation, for compound **1** (method of ionization (+)).

Compound **2**: 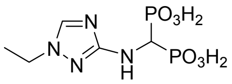

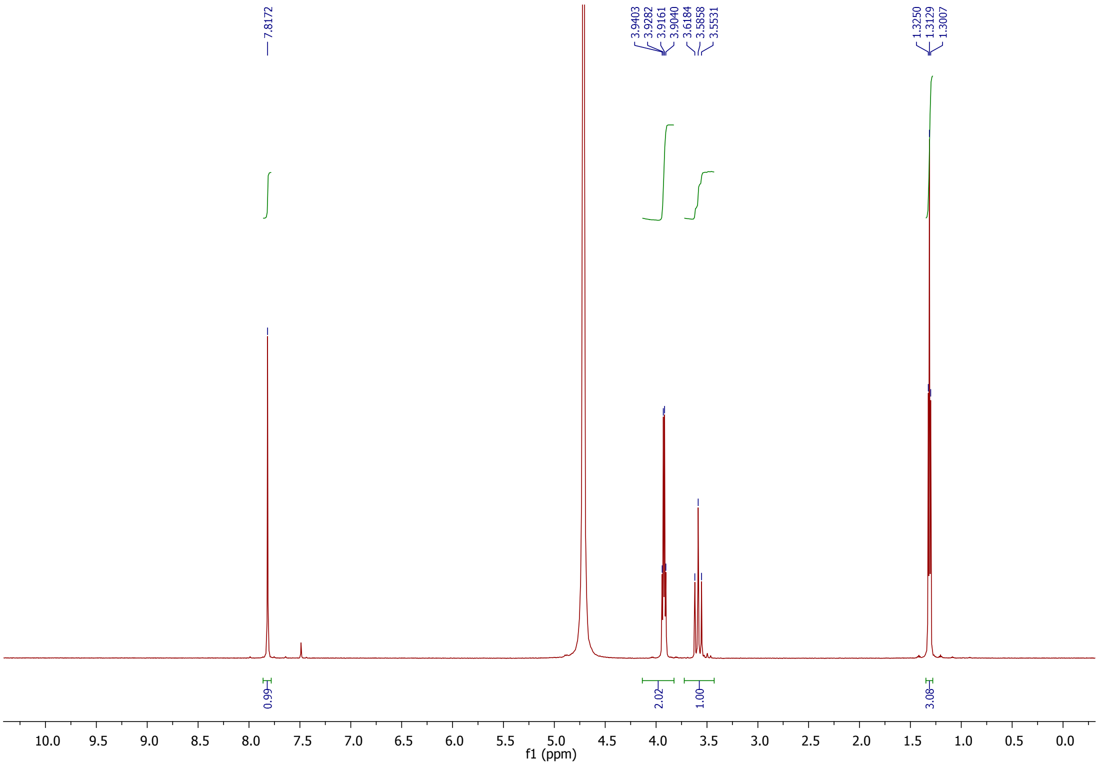

Figure S8. ^{1}H-NMR spectrum of compound **2.**

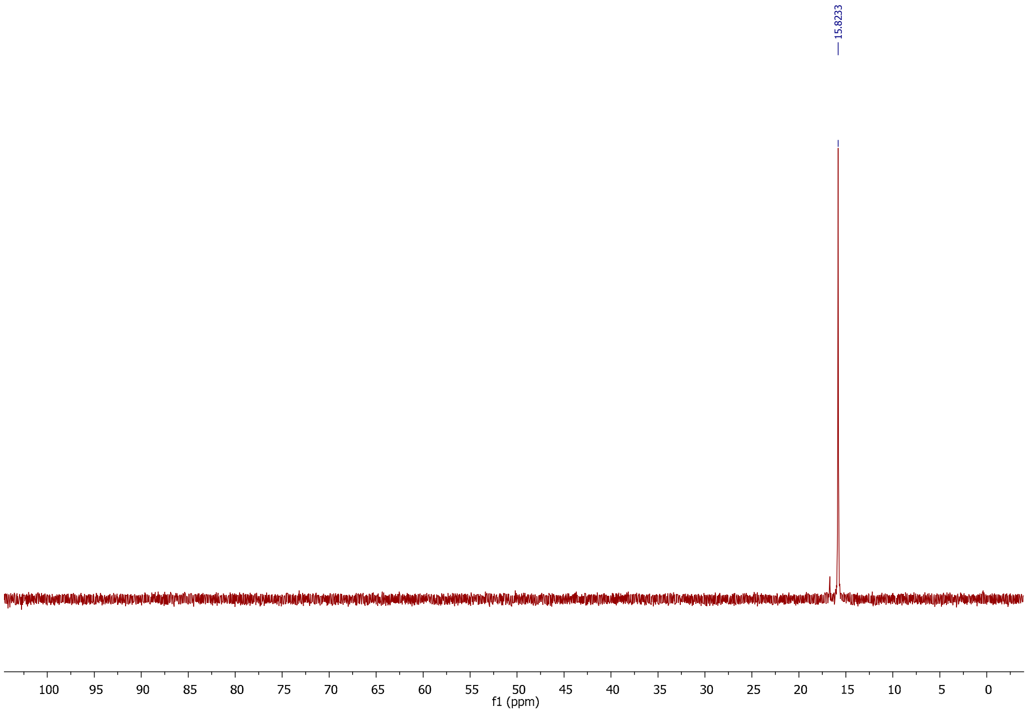

Figure S9. ^{31}P-NMR spectrum of compound **2**.

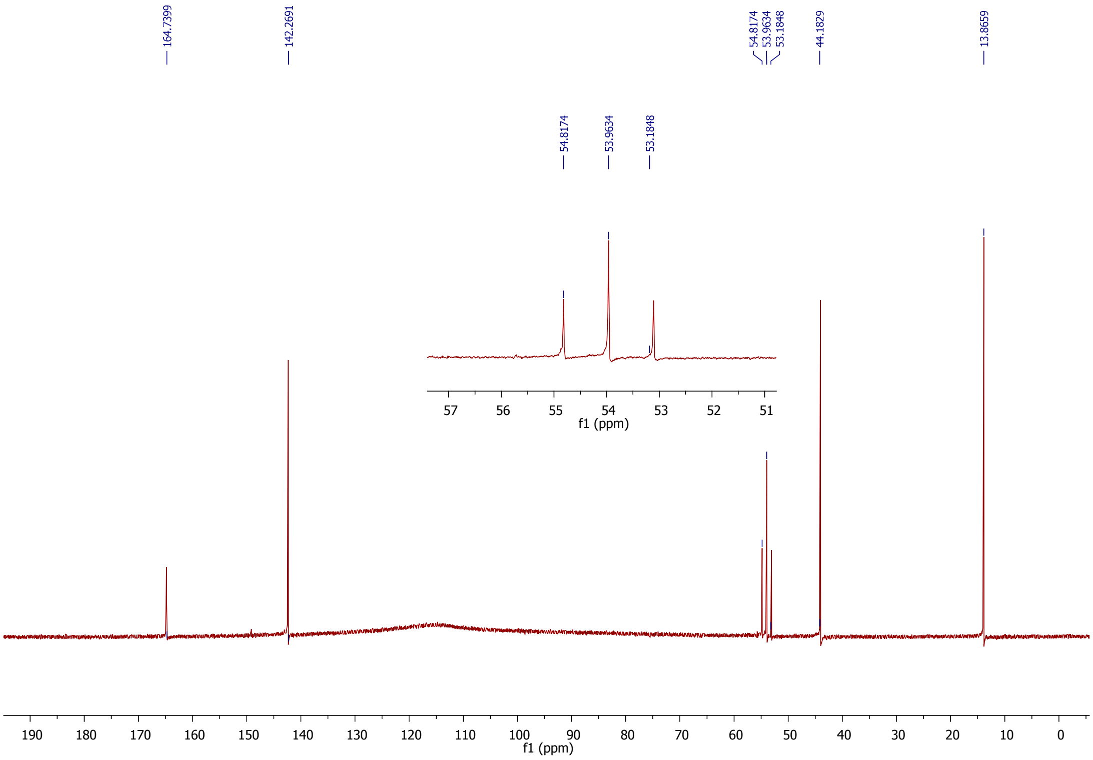

Figure S10. ^{13}C-NMR spectrum of compound **2**.

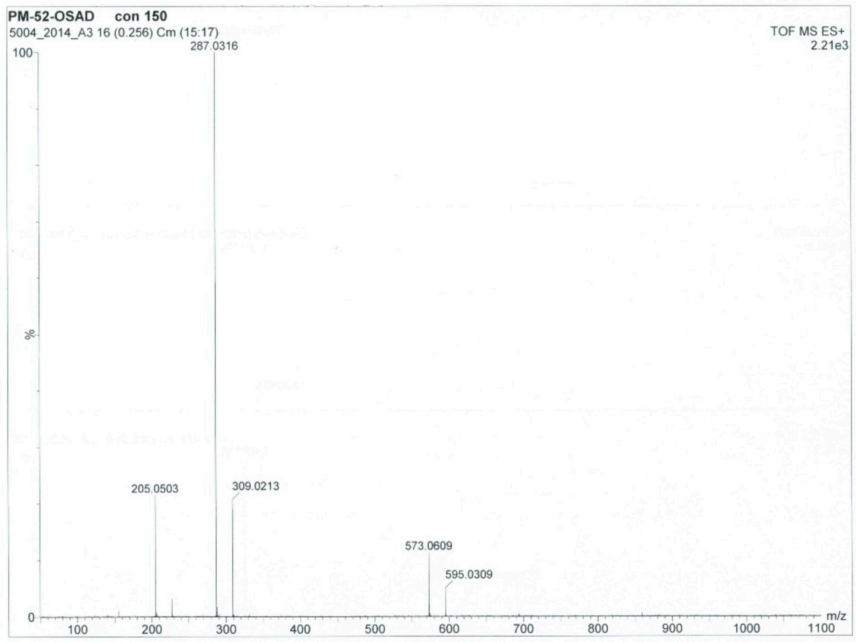

Figure S11. HRMS spectrum of compound **2**.

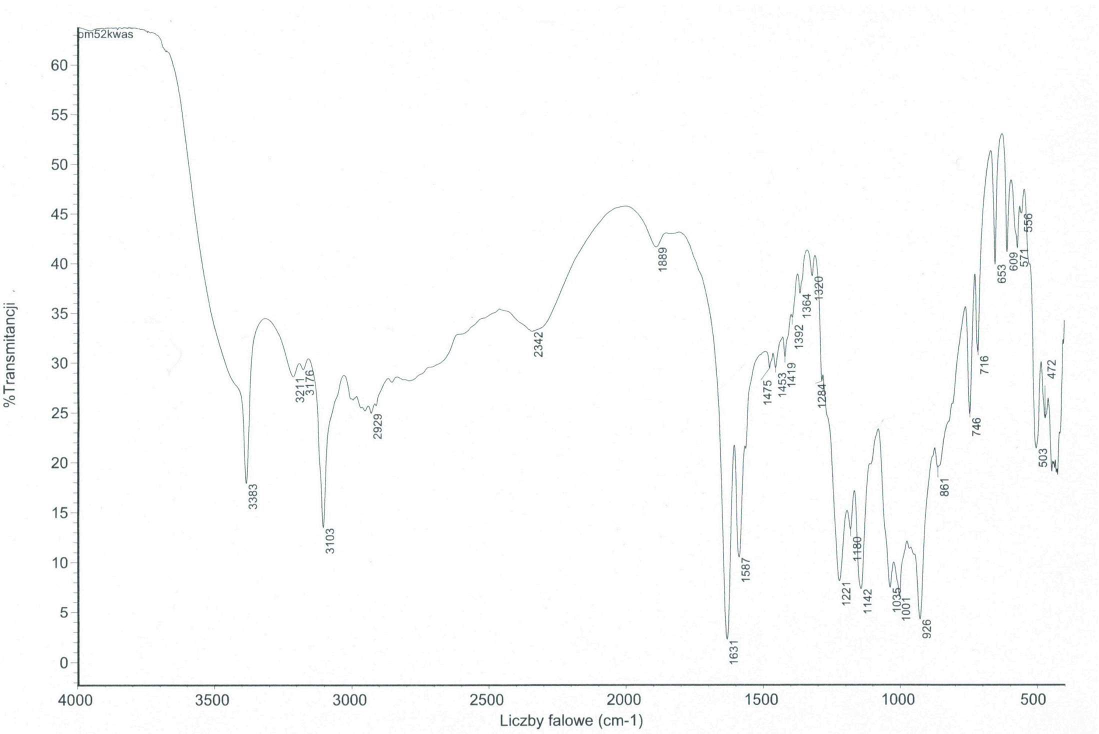

Figure S12. IR spectrum of compound **2.**

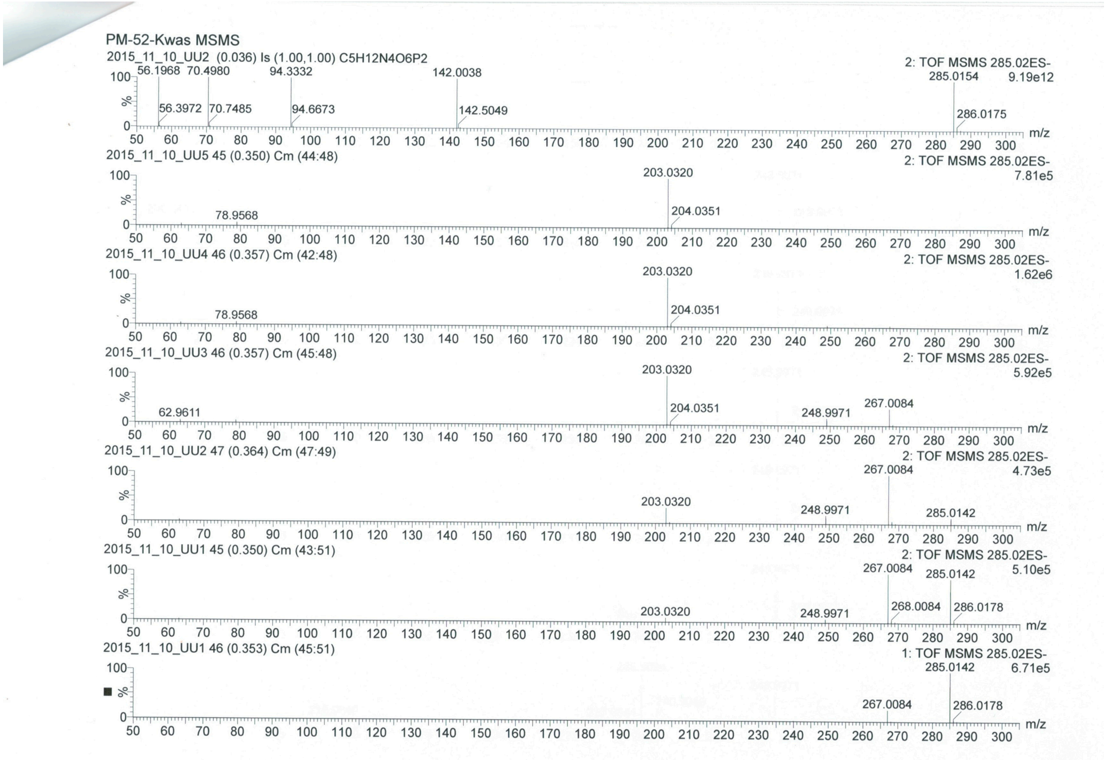

Figure S13. MS spectrum with fragmentation, for compound **2** (method of ionization (−)).

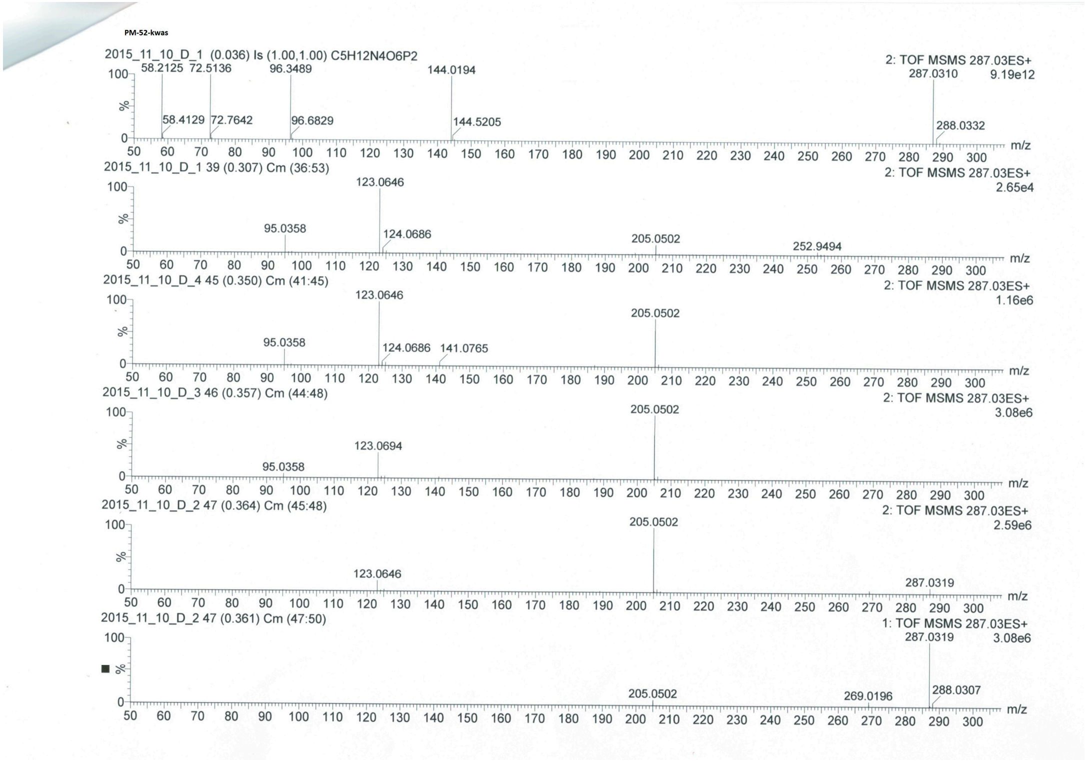

Figure S14. MS spectrum with fragmentation, for compound **2** (method of ionization (+)).

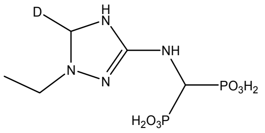

Compound **2** after prolonged (several days) storage in D_2O/NaOD solution:

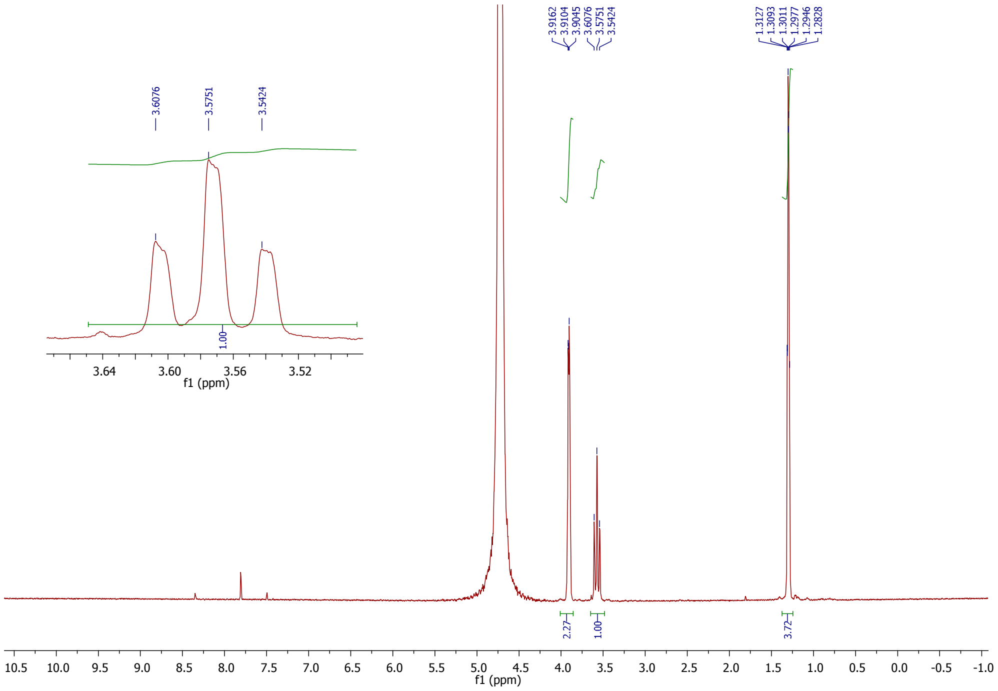

Figure S15. ^{1}H-NMR spectrum of compound **2** after prolonged (several days) storage in D2O/NaOD solution.

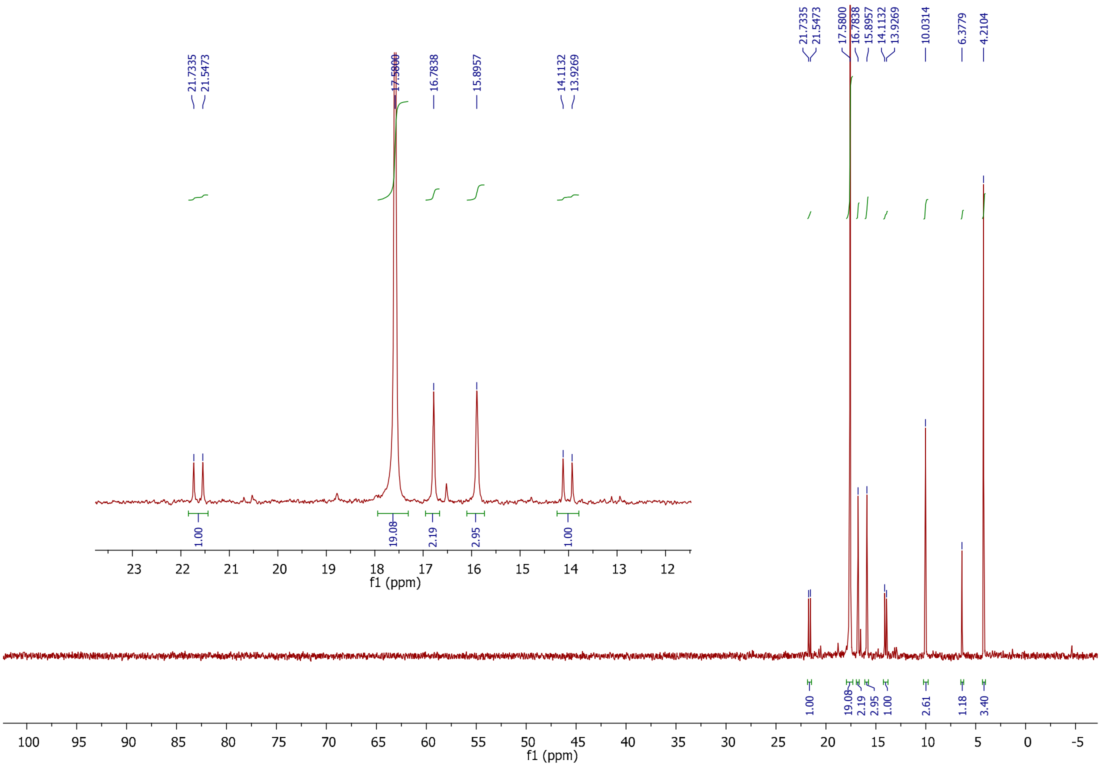

Figure S16. ^{31}P-NMR spectrum of a representative example of crude reaction mixture.

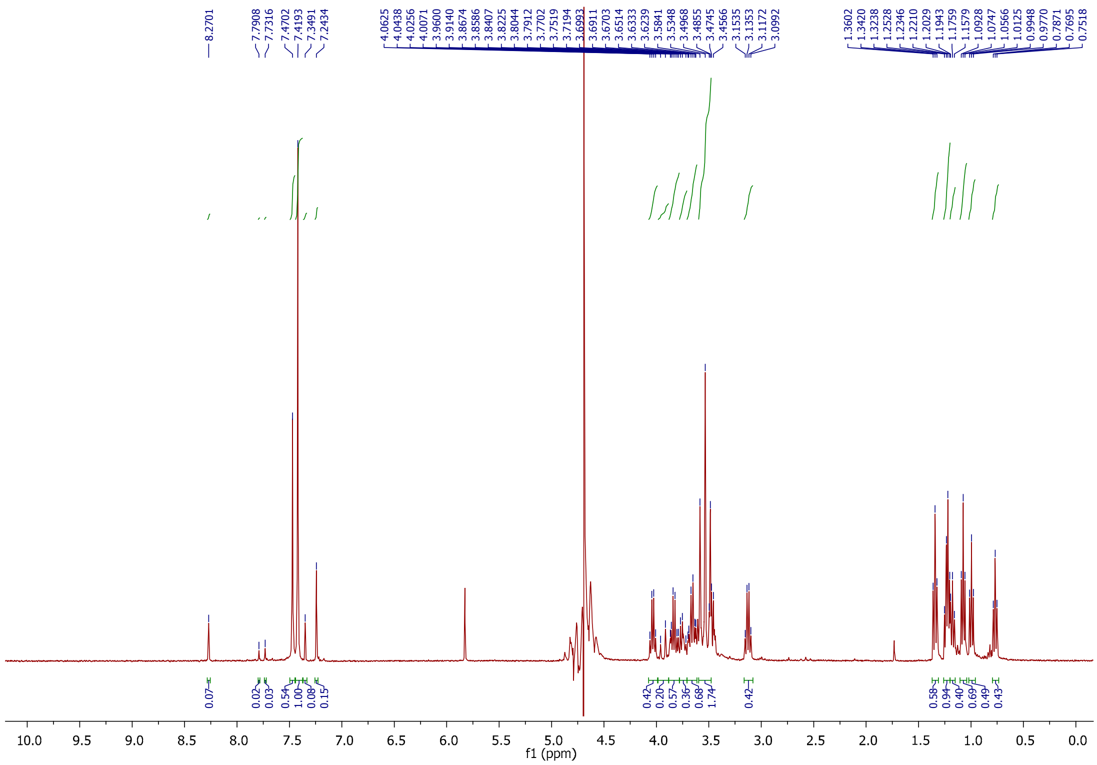

Figure S17. ^{1}H-NMR spectrum of a representative example of crude reaction mixture.

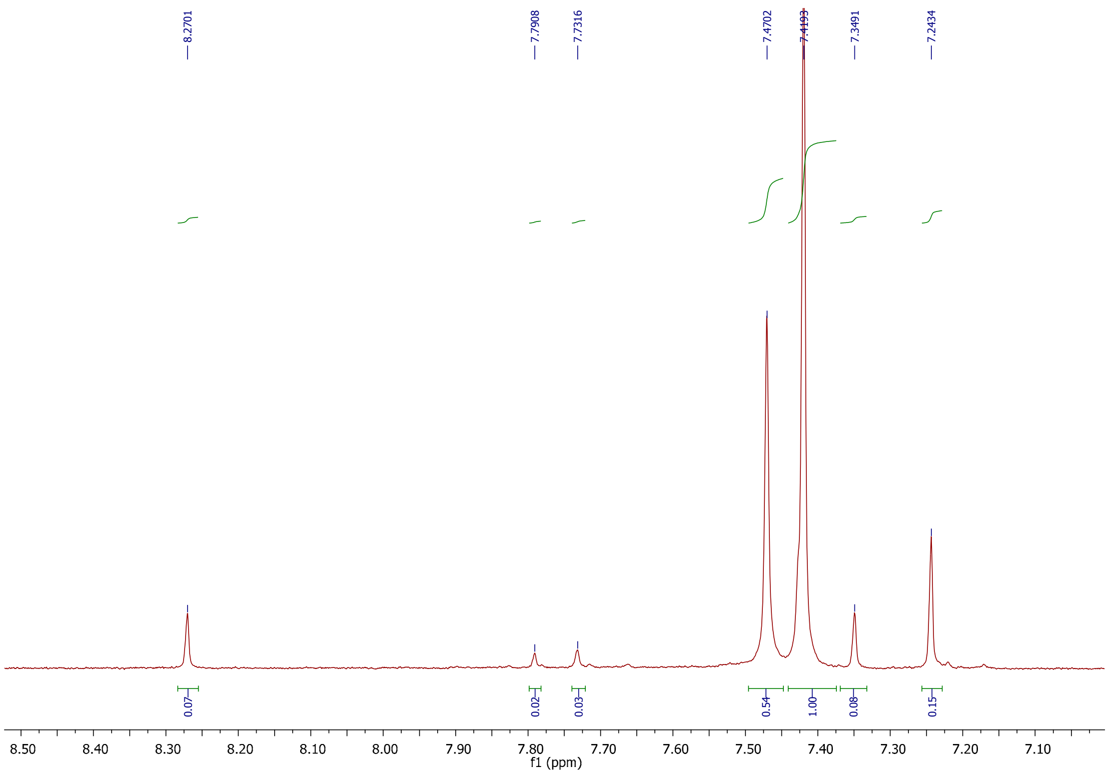

Figure S18. ^{1}H-NMR spectrum of a representative example of crude reaction mixture (aromatic range).

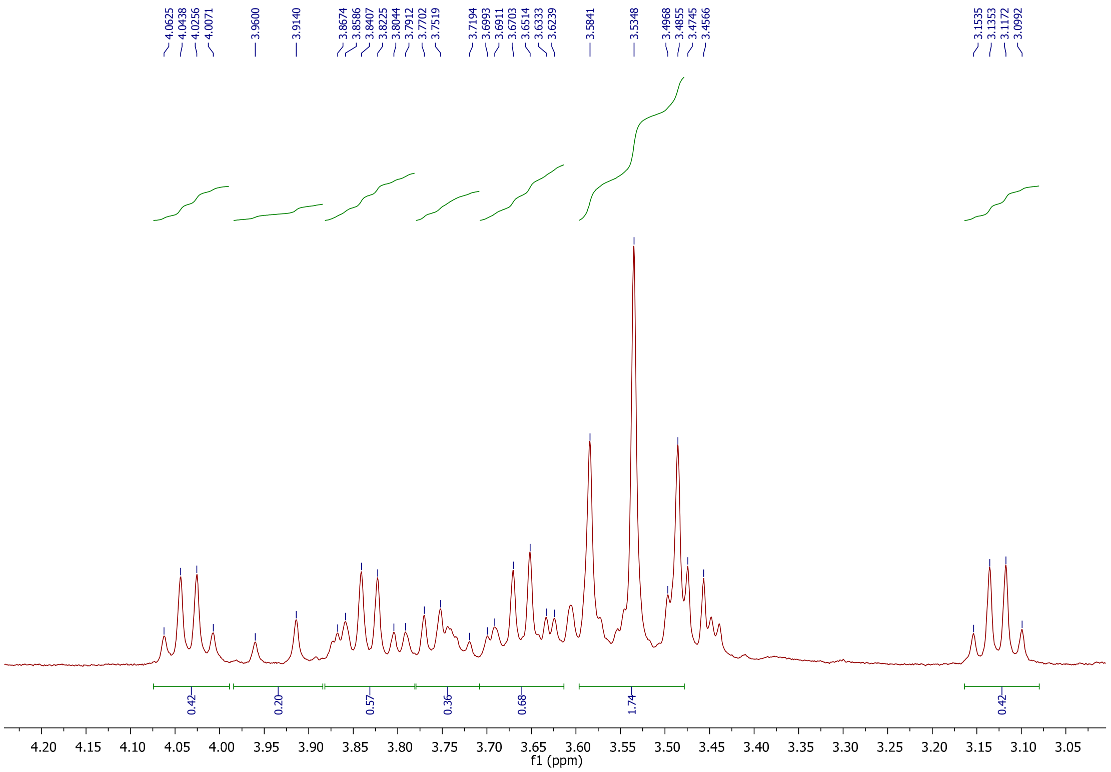

Figure S19. ^{1}H-NMR spectrum of a representative example of crude reaction mixture (aliphatic (ethyl) range).

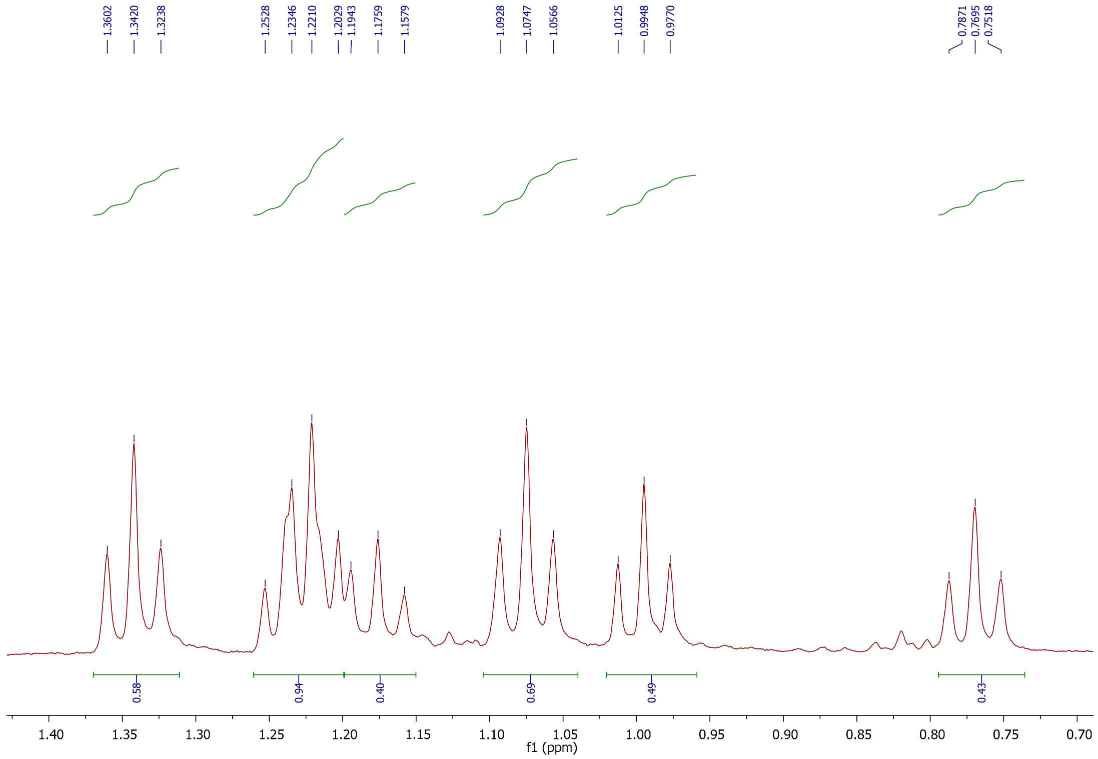

Figure S20. ^{1}H-NMR spectrum of a representative example of crude reaction mixture (aliphatic (methyl) range).

H-P correletion

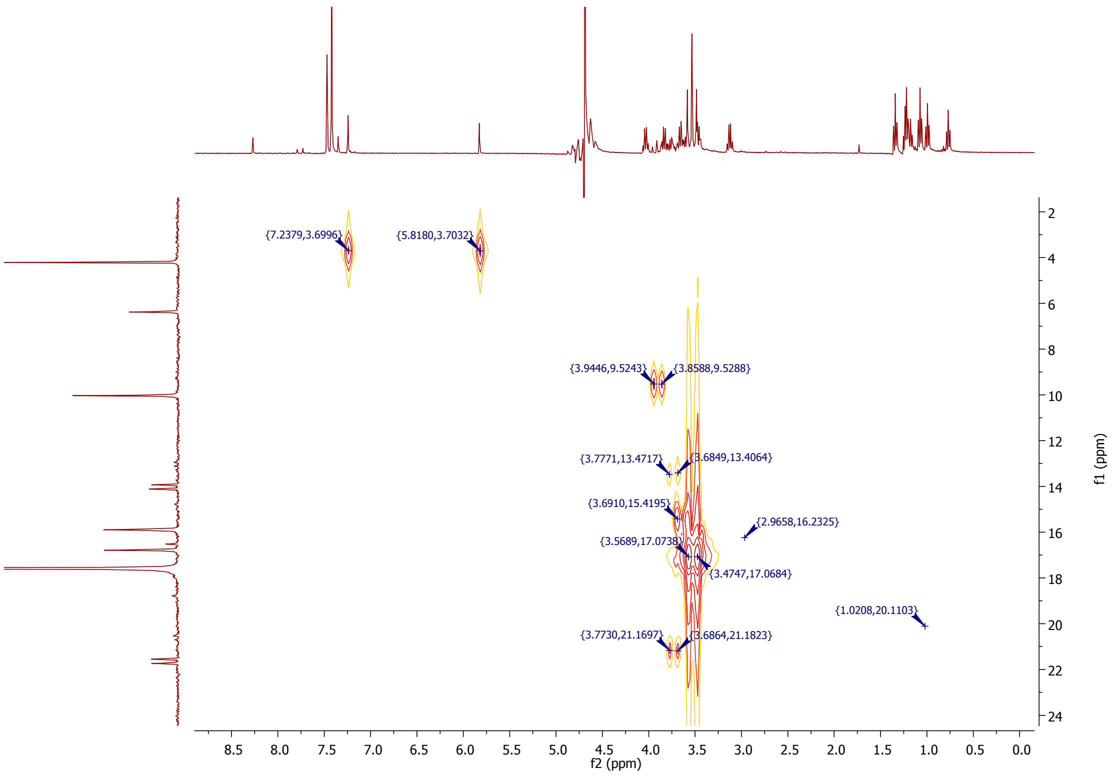

Figure S21. H-P correlation spectrum of a representative example of crude reaction mixture.

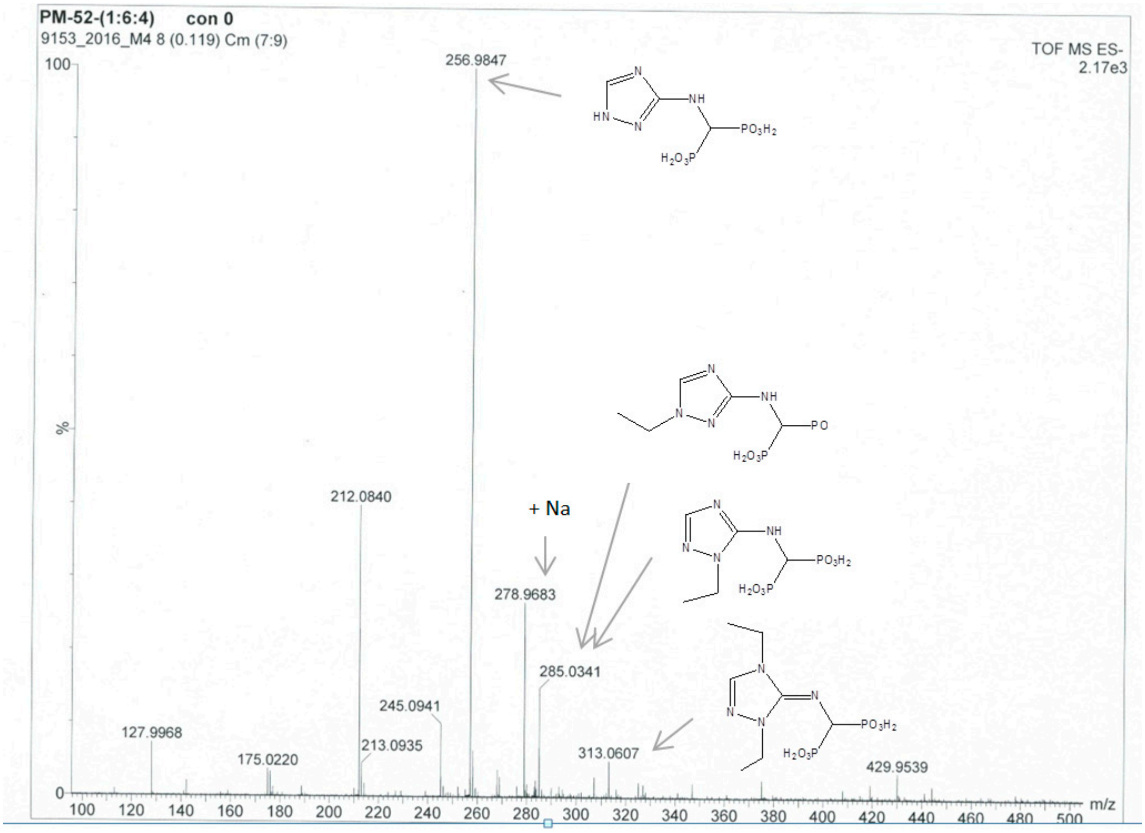

Figure S22. HRMS spectrum of a representative example of crude reaction mixture.

2. Spectroscopic Titrations

Compound 1 **Compound 2**

(A)

(B)

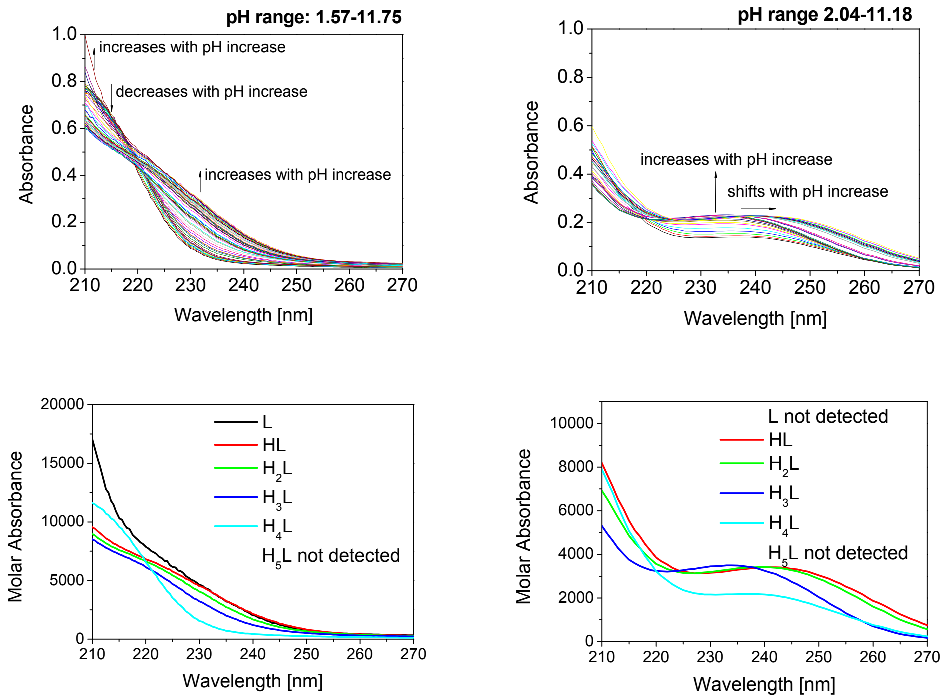

Figure S23. (**A**) Absorption spectrophotometric titration vs. pH of free compounds plotted in chosen pH values; (**B**) electronic spectra of species calculated in HypSpec.

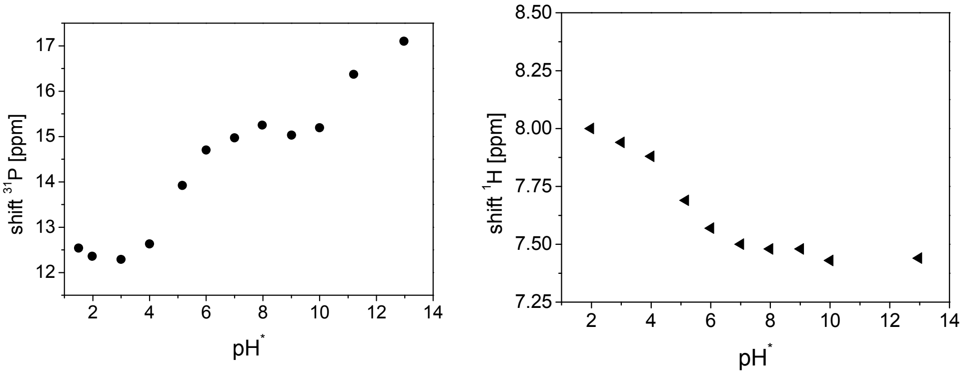

(**A**) (**B**)

Figure S24. ^{31}P (**A**) and $^{1}H_{aromatic}$ (**B**) NMR titration curves as a function of pH performed for compound **1**. Concentration of the compound: [L] = 4 × 10^{-2}. * not corrected for D_2O.

3. Relevant Crystallographic Data for the Molecules and the Full Geometrical Information

Table S1. Crystal data and structure refinement for compound **2**.

Identification Code	121_a
Empirical formula	C10 H30 Ca N8 O16 P4
Formula weight	682.38
Temperature	100.0 (1) K
Wavelength	0.71073 A
Crystal system, space group	Triclinic, P-1
Unit cell dimensions	*a* = 10.6815 (4) A, α = 60.518 (5)deg<br>*b* = 12.5015 (6) A, β = 66.986 (4)deg<br>*c* = 12.6155 (5) A, γ = 69.072 (4)deg
Volume	1320.90 (12) A^3
Z, Calculated density	2, 1.716 Mg/m^3
Absorption coefficient	0.566 mm^{-1}
F(000)	708
Crystal size	0.2 × 0.1 × 0.1 mm
Theta range for data collection	3.132°–25.999°
Limiting indices	$-13 \leq h \leq 13$, $-12 \leq k \leq 15$, $-15 \leq l \leq 15$
Reflections collected/unique	8995/5080 [R(int) = 0.0797]
Completeness to θ = 25.242	98.40%
Refinement method	Full-matrix least-squares on F^2
Data/restraints/parameters	5080/64/420
Goodness-of-fit on F^2	1.033
Final R indices [I > 2σ(I)]	R1 = 0.0790, wR2 = 0.1307
R indices (all data)	R1 = 0.1673, wR2 = 0.1644
Extinction coefficient	n/a
Largest diff. peak and hole	0.656 and −0.766 e·A^{-3}

Table S2. Atomic coordinates (×10[4]) and equivalent isotropic displacement parameters (A^2 × 10^3) for 121_a (compound **2**).

	X	Y	Z	U(eq)
Ca(1)	12,706(1)	4111(1)	5360(1)	18(1)
P(10A)	10,846(2)	5748(2)	2879(2)	15(1)
P(10B)	15,703(2)	2146(2)	6301(2)	20(1)
P(14A)	11,386(2)	2806(2)	4194(2)	17(1)
P(14B)	16,320(2)	4155(2)	3564(2)	17(1)
N(1A)	11,402(6)	3004(5)	96(5)	19(1)
N(2A)	11,718(6)	3357(5)	830(5)	19(1)
N(4A)	9396(6)	3685(5)	1169(5)	17(1)
N(8A)	10,205(6)	4285(5)	2266(5)	18(1)
N(1B)	19,244(6)	71(5)	2852(5)	22(1)
N(2B)	18,126(6)	787(5)	3410(5)	21(1)
N(4B)	20,151(6)	848(5)	3515(5)	17(1)
N(8B)	18,069(5)	1971(5)	4441(5)	18(1)
O(11A)	9320(5)	6216(4)	3109(4)	19(1)
O(12A)	11,548(5)	6653(5)	1518(4)	27(1)
O(13A)	11,477(5)	5609(4)	3841(4)	20(1)
O(15A)	11,735(5)	1713(4)	3864(5)	24(1)
O(16A)	12,406(5)	2785(4)	4751(4)	21(1)
O(17A)	9844(4)	2821(4)	5084(4)	18(1)
O(11B)	16,299(5)	777(5)	7072(4)	31(1)
O(12B)	14,188(5)	2466(4)	6465(4)	22(1)
O(13B)	16,216(5)	2902(5)	6688(5)	30(1)
O(15B)	16,744(5)	4191(5)	2223(4)	23(1)
O(16B)	14,783(5)	4729(4)	3883(4)	20(1)
O(17B)	17,268(5)	4704(4)	3690(4)	20(1)
O(18)	15,481(9)	−772(7)	7014(8)	92(3)
O(19)	13,343(10)	7763(9)	1028(10)	102(3)
O(20)	4590(18)	5097(18)	985(13)	81(5)
O(21)	4970(30)	3290(30)	1580(30)	68(7)
O(22)	4508(8)	2924(8)	794(9)	54(2)
O(23)	4198(16)	466(11)	5186(13)	61(4)
C(3A)	10,463(7)	3773(6)	1447(6)	18(2)
C(5A)	10,062(8)	3200(6)	309(6)	22(2)
C(6A)	12,529(8)	2531(6)	-809(6)	24(2)
C(7A)	13,346(8)	1271(7)	-157(7)	38(2)
C(9A)	11,267(7)	4244(6)	2756(6)	15(2)
C(3B)	18,736(7)	1228(6)	3807(6)	19(2)
C(5B)	20,413(8)	104(6)	2920(6)	22(2)
C(6B)	18,979(9)	−595(7)	2293(7)	36(2)
C(7B)	18,382(9)	295(8)	1193(8)	44(2)
C(9B)	16,581(7)	2479(6)	4633(6)	17(2)

U(eq) is defined as one third of the trace of the orthogonalized Uij tensor.

Table S3. Bond lengths [A] and angles [deg] for 121_a (compound **2**). Symmetry transformations used to generate equivalent atoms: #1 –x + 2, −y + 1, −z + 1; #2 –x + 3, −y + 1, −z + 1; #3 –x + 1, −y, −z + 1.

Atoms	Distance
Ca(1)-O(11A) #1	2.273(5)
Ca(1)-O(12B)	2.292(5)
Ca(1)-O(16A)	2.295(5)
Ca(1)-O(17B) #2	2.331(5)
Ca(1)-O(16B)	2.359(5)
Ca(1)-O(13A)	2.363(5)
Ca(1)-P(10B)	3.489(2)
Ca(1)-P(14A)	3.528(2)
Ca(1)-P(10A) #1	3.561(2)
P(10A)-O(11A)	1.483(5)
P(10A)-O(13A)	1.518(5)
P(10A)-O(12A)	1.574(5)
P(10A)-C(9A)	1.841(6)
P(10A)-Ca(1) #1	3.561(2)
P(10B)-O(12B)	1.476(5)
P(10B)-O(11B)	1.533(5)
P(10B)-O(13B)	1.571(5)
P(10B)-C(9B)	1.832(6)
P(14A)-O(16A)	1.498(5)
P(14A)-O(15A)	1.501(5)
P(14A)-O(17A)	1.589(5)
P(14A)-C(9A)	1.830(6)
P(14B)-O(17B)	1.502(5)
P(14B)-O(16B)	1.516(5)
P(14B)-O(15B)	1.552(4)
P(14B)-C(9B)	1.839(7)
N(1A)-C(5A)	1.302(9)
N(1A)-N(2A)	1.385(7)
N(1A)-C(6A)	1.478(8)
N(2A)-C(3A)	1.326(8)
N(4A)-C(5A)	1.337(8)
N(4A)-C(3A)	1.368(8)
N(8A)-C(3A)	1.362(8)
N(8A)-C(9A)	1.465(8)
N(8A)-H(8AA)	0.8600
N(1B)-C(5B)	1.301(9)
N(1B)-N(2B)	1.386(7)
N(1B)-C(6B)	1.474(9)
N(2B)-C(3B)	1.327(8)
N(4B)-C(5B)	1.358(8)
N(4B)-C(3B)	1.368(8)
N(8B)-C(3B)	1.351(8)
N(8B)-C(9B)	1.456(8)
N(8B)-H(8BA)	0.8600
O(11A)-Ca(1) #1	2.273(5)
O(12A)-H(12A)	0.837(10)
O(15A)-H(15A)	0.841(10)
O(17A)-H(17A)	0.840(10)
O(11B)-H(11B)	1.03(9)

O(13B)-H(13B)	0.88(8)
O(15B)-H(15B)	0.82(7)
O(17B)-Ca(1) #2	2.331(5)
O(18)-H(18A)	0.843(10)
O(18)-H(18B)	0.841(10)
O(19)-H(19A)	0.848(10)
O(19)-H(19B)	0.854(10)
O(20)-H(20A)	0.839(10)
O(20)-H(20B)	0.840(10)
O(20)-H(21D)	1.23(18)
O(21)-O(22)	1.56(3)
O(21)-H(21C)	0.839(11)
O(21)-H(21D)	0.840(10)
O(22)-H(22A)	0.841(10)
O(22)-H(22B)	0.844(10)
O(23)-O(23) #3	1.73(3)
O(23)-H(23A)	0.840(10)
O(23)-H(23B)	0.842(10)
C(5A)-H(5AB)	0.93
C(6A)-C(7A)	1.491(9)
C(6A)-H(6AA)	0.9700
C(6A)-H(6AB)	0.9700
C(7A)-H(7AA)	0.9600
C(7A)-H(7AB)	0.9600
C(7A)-H(7AC)	0.9600
C(9A)-H(9AA)	0.9800
C(5B)-H(5BA)	0.9300
C(6B)-C(7B)	1.496(10)
C(6B)-H(6BA)	0.9700
C(6B)-H(6BB)	0.9700
C(7B)-H(7BA)	0.9600
C(7B)-H(7BB)	0.9600
C(7B)-H(7BC)	0.9600
C(9B)-H(9BA)	0.9800
O(11A) #1-Ca(1)-O(12B)	98.32(16)
O(11A) #1-Ca(1)-O(16A)	87.76(16)
O(12B)-Ca(1)-O(16A)	88.68(16)
O(11A) #1-Ca(1)-O(17B) #2	82.68(16)
O(12B)-Ca(1)-O(17B) #2	89.53(16)
O(16A)-Ca(1)-O(17B) #2	169.92(17)
O(11A) #1-Ca(1)-O(16B)	169.68(16)
O(12B)-Ca(1)-O(16B)	83.59(16)
O(16A)-Ca(1)-O(16B)	102.44(17)
O(17B) #2-Ca(1)-O(16B)	87.20(16)
O(11A) #1-Ca(1)-O(13A)	90.91(16)
O(12B)-Ca(1)-O(13A)	165.95(16)
O(16A)-Ca(1)-O(13A)	81.08(16)
O(17B) #2-Ca(1)-O(13A)	102.20(16)
O(16B)-Ca(1)-O(13A)	89.22(16)
O(11A) #1-Ca(1)-P(10B)	115.07(12)
O(12B)-Ca(1)-P(10B)	17.58(12)
O(16A)-Ca(1)-P(10B)	94.50(12)

O(17B) #2-Ca(1)-P(10B)	86.69(12)
O(16B)-Ca(1)-P(10B)	66.14(11)
O(13A)-Ca(1)-P(10B)	153.56(12)
O(11A) #1-Ca(1)-P(14A)	80.88(12)
O(12B)-Ca(1)-P(14A)	105.20(12)
O(16A)-Ca(1)-P(14A)	17.19(11)
O(17B) #2-Ca(1)-P(14A)	159.30(12)
O(16B)-Ca(1)-P(14A)	108.50(12)
O(13A)-Ca(1)-P(14A)	65.68(12)
P(10B)-Ca(1)-P(14A)	111.69(5)
O(11A) #1-Ca(1)-P(10A) #1	14.86(12)
O(12B)-Ca(1)-P(10A) #1	113.13(12)
O(16A)-Ca(1)-P(10A) #1	87.00(12)
O(17B) #2-Ca(1)-P(10A) #1	84.59(12)
O(16B)-Ca(1)-P(10A) #1	161.24(12)
O(13A)-Ca(1)-P(10A) #1	76.12(12)
P(10B)-Ca(1)-P(10A) #1	129.94(6)
P(14A)-Ca(1)-P(10A) #1	76.34(5)
O(11A)-P(10A)-O(13A)	115.8(3)
O(11A)-P(10A)-O(12A)	107.8(3)
O(13A)-P(10A)-O(12A)	110.2(3)
O(11A)-P(10A)-C(9A)	108.2(3)
O(13A)-P(10A)-C(9A)	110.5(3)
O(12A)-P(10A)-C(9A)	103.7(3)
O(11A)-P(10A)-Ca(1) #1	23.15(18)
O(13A)-P(10A)-Ca(1) #1	99.72(18)
O(12A)-P(10A)-Ca(1) #1	130.8(2)
C(9A)-P(10A)-Ca(1) #1	101.0(2)
O(12B)-P(10B)-O(11B)	115.7(3)
O(12B)-P(10B)-O(13B)	114.2(3)
O(11B)-P(10B)-O(13B)	103.0(3)
O(12B)-P(10B)-C(9B)	108.5(3)
O(11B)-P(10B)-C(9B)	107.3(3)
O(13B)-P(10B)-C(9B)	107.7(3)
O(12B)-P(10B)-Ca(1)	27.97(18)
O(11B)-P(10B)-Ca(1)	142.1(2)
O(13B)-P(10B)-Ca(1)	105.2(2)
C(9B)-P(10B)-Ca(1)	87.5(2)
O(16A)-P(14A)-O(15A)	114.4(3)
O(16A)-P(14A)-O(17A)	113.9(3)
O(15A)-P(14A)-O(17A)	105.3(3)
O(16A)-P(14A)-C(9A)	109.7(3)
O(15A)-P(14A)-C(9A)	107.8(3)
O(17A)-P(14A)-C(9A)	105.2(3)
O(16A)-P(14A)-Ca(1)	26.93(17)
O(15A)-P(14A)-Ca(1)	141.2(2)
O(17A)-P(14A)-Ca(1)	100.34(17)
C(9A)-P(14A)-Ca(1)	92.8(2)
O(17B)-P(14B)-O(16B)	116.1(3)
O(17B)-P(14B)-O(15B)	111.7(3)
O(16B)-P(14B)-O(15B)	108.2(3)
O(17B)-P(14B)-C(9B)	106.6(3)

O(16B)-P(14B)-C(9B)	108.4(3)
O(15B)-P(14B)-C(9B)	105.3(3)
C(5A)-N(1A)-N(2A)	110.6(5)
C(5A)-N(1A)-C(6A)	129.2(6)
N(2A)-N(1A)-C(6A)	120.1(6)
C(3A)-N(2A)-N(1A)	101.7(5)
C(5A)-N(4A)-C(3A)	102.8(6)
C(3A)-N(8A)-C(9A)	124.1(6)
C(3A)-N(8A)-H(8AA)	117.9
C(9A)-N(8A)-H(8AA)	117.9
C(5B)-N(1B)-N(2B)	111.3(5)
C(5B)-N(1B)-C(6B)	129.8(6)
N(2B)-N(1B)-C(6B)	118.9(6)
C(3B)-N(2B)-N(1B)	102.5(5)
C(5B)-N(4B)-C(3B)	104.1(6)
C(3B)-N(8B)-C(9B)	121.3(6)
C(3B)-N(8B)-H(8BA)	119.4
C(9B)-N(8B)-H(8BA)	119.4
P(10A)-O(11A)-Ca(1) #1	142.0(3)
P(10A)-O(12A)-H(12A)	109.5
P(10A)-O(13A)-Ca(1)	142.6(3)
P(14A)-O(15A)-H(15A)	109.5
P(14A)-O(16A)-Ca(1)	135.9(3)
P(14A)-O(17A)-H(17A)	109.5
P(10B)-O(11B)-H(11B)	109.5
P(10B)-O(12B)-Ca(1)	134.4(3)
P(10B)-O(13B)-H(13B)	109.5
P(14B)-O(15B)-H(15B)	109.5
P(14B)-O(16B)-Ca(1)	138.6(3)
P(14B)-O(17B)-Ca(1) #2	142.5(3)
H(18A)-O(18)-H(18B)	105(3)
H(19A)-O(19)-H(19B)	103(3)
H(20A)-O(20)-H(20B)	106(3)
H(20A)-O(20)-H(21D)	157(10)
H(20B)-O(20)-H(21D)	51(10)
O(22)-O(21)-H(21C)	109(10)
O(22)-O(21)-H(21D)	120(10)
H(21C)-O(21)-H(21D)	106(3)
O(21)-O(22)-H(22A)	117(9)
O(21)-O(22)-H(22B)	106(8)
H(22A)-O(22)-H(22B)	105(3)
O(23) #3-O(23)-H(23A)	117(10)
O(23) #3-O(23)-H(23B)	64(10)
H(23A)-O(23)-H(23B)	105(3)
N(2A)-C(3A)-N(8A)	124.8(7)
N(2A)-C(3A)-N(4A)	114.2(6)
N(8A)-C(3A)-N(4A)	120.9(6)
N(1A)-C(5A)-N(4A)	110.7(6)
N(1A)-C(5A)-H(5AB)	124.7
N(4A)-C(5A)-H(5AB)	124.7
N(1A)-C(6A)-C(7A)	112.0(6)
N(1A)-C(6A)-H(6AA)	109.2

C(7A)-C(6A)-H(6AA)	109.2
N(1A)-C(6A)-H(6AB)	109.2
C(7A)-C(6A)-H(6AB)	109.2
H(6AA)-C(6A)-H(6AB)	107.9
C(6A)-C(7A)-H(7AA)	109.5
C(6A)-C(7A)-H(7AB)	109.5
H(7AA)-C(7A)-H(7AB)	109.5
C(6A)-C(7A)-H(7AC)	109.5
H(7AA)-C(7A)-H(7AC)	109.5
H(7AB)-C(7A)-H(7AC)	109.5
N(8A)-C(9A)-P(14A)	108.9(4)
N(8A)-C(9A)-P(10A)	107.0(4)
P(14A)-C(9A)-P(10A)	117.4(3)
N(8A)-C(9A)-H(9AA)	107.7
P(14A)-C(9A)-H(9AA)	107.7
P(10A)-C(9A)-H(9AA)	107.7
N(2B)-C(3B)-N(8B)	125.3(6)
N(2B)-C(3B)-N(4B)	113.1(6)
N(8B)-C(3B)-N(4B)	121.7(6)
N(1B)-C(5B)-N(4B)	109.0(6)
N(1B)-C(5B)-H(5BA)	125.5
N(4B)-C(5B)-H(5BA)	125.5
N(1B)-C(6B)-C(7B)	111.9(6)
N(1B)-C(6B)-H(6BA)	109.2
C(7B)-C(6B)-H(6BA)	109.2
N(1B)-C(6B)-H(6BB)	109.2
C(7B)-C(6B)-H(6BB)	109.2
H(6BA)-C(6B)-H(6BB)	107.9
C(6B)-C(7B)-H(7BA)	109.5
C(6B)-C(7B)-H(7BB)	109.5
H(7BA)-C(7B)-H(7BB)	109.5
C(6B)-C(7B)-H(7BC)	109.5
H(7BA)-C(7B)-H(7BC)	109.5
H(7BB)-C(7B)-H(7BC)	109.5
N(8B)-C(9B)-P(10B)	111.3(5)
N(8B)-C(9B)-P(14B)	108.2(4)
P(10B)-C(9B)-P(14B)	114.5(3)
N(8B)-C(9B)-H(9BA)	107.5
P(10B)-C(9B)-H(9BA)	107.5
P(14B)-C(9B)-H(9BA)	107.5

Table S4. Anisotropic displacement parameters ($A^2 \times 10^3$) for 121_a (compound **2**).

	U11	U22	U33	U23	U13	U12
Ca(1)	16(1)	16(1)	18(1)	−9(1)	−3(1)	1(1)
P(10A)	17(1)	14(1)	13(1)	−6(1)	−2(1)	−2(1)
P(10B)	22(1)	21(1)	13(1)	−5(1)	−4(1)	−3(1)
P(14A)	17(1)	14(1)	19(1)	−10(1)	−3(1)	3(1)
P(14B)	19(1)	15(1)	11(1)	−6(1)	−3(1)	1(1)
N(1A)	28(4)	11(3)	10(3)	−4(2)	−1(3)	−1(3)
N(2A)	21(3)	15(3)	12(3)	−5(2)	0(3)	0(3)
N(4A)	28(3)	12(3)	8(3)	−3(2)	−2(3)	−6(3)
N(8A)	17(3)	18(3)	15(3)	−10(3)	−1(3)	1(3)

N(1B)	37(4)	4(3)	16(3)	−3(2)	−5(3)	1(3)
N(2B)	30(4)	11(3)	14(3)	−4(3)	−2(3)	−2(3)
N(4B)	16(3)	11(3)	10(3)	1(2)	−2(2)	3(2)
N(8B)	13(3)	20(3)	22(3)	−15(3)	−6(3)	5(2)
O(11A)	19(3)	16(2)	22(3)	−10(2)	−5(2)	1(2)
O(12A)	33(3)	29(3)	17(3)	−8(2)	2(2)	−15(3)
O(13A)	20(3)	16(3)	21(3)	−10(2)	−4(2)	1(2)
O(15A)	22(3)	25(3)	28(3)	−15(2)	−9(2)	0(2)
O(16A)	23(3)	16(3)	19(3)	−9(2)	−7(2)	4(2)
O(17A)	23(3)	15(3)	18(3)	−12(2)	−3(2)	−4(2)
O(11B)	29(3)	28(3)	20(3)	−6(2)	−4(2)	4(2)
O(12B)	22(3)	18(3)	16(3)	−3(2)	−1(2)	−3(2)
O(13B)	40(3)	27(3)	24(3)	−11(3)	−6(3)	−9(3)
O(15B)	25(3)	25(3)	10(3)	−2(2)	−1(2)	−5(2)
O(16B)	22(3)	16(3)	12(2)	−2(2)	−1(2)	−1(2)
O(17B)	19(3)	19(3)	19(3)	−8(2)	−2(2)	−1(2)
O(18)	146(8)	39(4)	108(7)	−10(4)	−72(6)	−27(5)
O(19)	115(7)	99(6)	106(7)	−34(6)	−35(6)	−44(6)
O(20)	80(11)	134(12)	43(8)	−23(9)	−11(7)	−61(9)
O(21)	78(15)	77(15)	63(14)	−35(12)	−5(11)	−34(12)
O(22)	43(5)	56(6)	57(6)	−18(5)	−10(4)	−14(4)
O(23)	69(10)	36(8)	84(10)	−37(7)	−33(9)	15(7)
C(3A)	24(4)	12(4)	11(4)	−2(3)	−3(3)	−4(3)
C(5A)	33(5)	16(4)	15(4)	−5(3)	−7(3)	−5(3)
C(6A)	35(5)	18(4)	13(4)	−10(3)	1(3)	−3(3)
C(7A)	38(5)	27(5)	21(4)	−7(4)	7(4)	4(4)
C(9A)	19(4)	11(3)	13(4)	−5(3)	−5(3)	−1(3)
C(3B)	29(4)	8(3)	13(4)	4(3)	−6(3)	−7(3)
C(5B)	24(4)	13(4)	18(4)	−7(3)	−4(3)	7(3)
C(6B)	56(6)	26(4)	26(5)	−17(4)	−2(4)	−9(4)
C(7B)	54(6)	41(5)	36(5)	−22(4)	−9(5)	−4(5)
C(9B)	16(4)	13(3)	18(4)	−8(3)	−4(3)	5(3)

The anisotropic displacement factor exponent takes the form: $-2 pi^2$ [$h^2 a^{*2}$ U11 + ... + 2 h k a* b* U12].

Table S5. Hydrogen coordinates (×10^4) and isotropic displacement parameters ($A^2 \times 10^3$) for 121_a (compound **2**).

	X	Y	Z	U(eq)
H(8AA)	9367	4655	2511	21
H(8BA)	18,533	2148	4738	21
H(12A)	12,250(50)	6790(60)	1534(16)	40
H(15A)	11,020(20)	1640(40)	3800(70)	36
H(17A)	9680(20)	3250(50)	5480(50)	26
H(11B)	15,940(70)	190(50)	6950(60)	47
H(13B)	15,800(70)	3700(70)	6390(70)	45
H(15B)	17,590(80)	4080(70)	1950(40)	35
H(18A)	14,700(60)	−450(80)	7380(100)	110
H(18B)	15,510(100)	−1551(17)	7400(90)	110
H(19A)	13,770(100)	8270(70)	950(110)	122
H(19B)	13,750(110)	7050(40)	1480(110)	122
H(20A)	4500(200)	5870(30)	540(150)	97
H(20B)	5250(150)	4760(150)	530(140)	97
H(21C)	5700(200)	2740(180)	1800(300)	82

H(21D)	5200(300)	3990(110)	1300(400)	82
H(22A)	3840(70)	2550(80)	1170(90)	65
H(22B)	4220(100)	3610(60)	230(80)	65
H(23A)	4200(190)	1150(80)	5160(130)	73
H(23B)	4500(200)	550(140)	4430(50)	73
H(5AB)	9628	3027	−84	26
H(6AA)	13,147	3119	−1339	29
H(6AB)	12,132	2478	−1349	29
H(7AA)	13,731	1317	388	58
H(7AB)	14,084	1008	−773	58
H(7AC)	12,747	676	336	58
H(9AA)	12,162	4200	2125	18
H(5BA)	21,287	−315	2611	26
H(6BA)	18,339	−1139	2930	43
H(6BB)	19,843	−1118	2019	43
H(7BA)	19,010	838	562	66
H(7BB)	17,506	789	1468	66
H(7BC)	18,245	−171	842	66
H(9BA)	16,208	2056	4377	21

Table S6. Torsion angles (°) for 121_a (compound **2**). Symmetry transformations used to generate equivalent atoms: #1 −x + 2, −y + 1, −z + 1; #2 −x + 3, −y + 1, −z + 1; #3 −x + 1, −y, −z + 1.

C(5A)-N(1A)-N(2A)-C(3A)	−0.7(7)
C(6A)-N(1A)-N(2A)-C(3A)	177.0(5)
C(5B)-N(1B)-N(2B)-C(3B)	−0.3(7)
C(6B)-N(1B)-N(2B)-C(3B)	179.5(6)
O(13A)-P(10A)-O(11A)-Ca(1) #1	−49.4(5)
O(12A)-P(10A)-O(11A)-Ca(1) #1	−173.2(4)
C(9A)-P(10A)-O(11A)-Ca(1) #	175.2(5)
O(11A)-P(10A)-O(13A)-Ca(1)	117.8(4)
O(12A)-P(10A)-O(13A)-Ca(1)	−119.6(4)
C(9A)-P(10A)-O(13A)-Ca(1)	−5.6(5)
Ca(1)#1-P(10A)-O(13A)-Ca(1)	100.2(4)
O(15A)-P(14A)-O(16A)-Ca(1)	175.0(3)
O(17A)-P(14A)-O(16A)-Ca(1)	−63.9(4)
C(9A)-P(14A)-O(16A)-Ca(1)	53.8(5)
O(11B)-P(10B)-O(12B)-Ca(1)	−164.0(3)
O(13B)-P(10B)-O(12B)-Ca(1)	76.7(4)
C(9B)-P(10B)-O(12B)-Ca(1)	−43.4(5)
O(17B)-P(14B)-O(16B)-Ca(1)	−117.4(4)
O(15B)-P(14B)-O(16B)-Ca(1)	116.2(4)
C(9B)-P(14B)-O(16B)-Ca(1)	2.4(5)
O(16B)-P(14B)-O(17B)-Ca(1) #2	19.5(5)
O(15B)-P(14B)-O(17B)-Ca(1) #2	144.2(4)
C(9B)-P(14B)-O(17B)-Ca(1) #2	−101.3(4)
N(1A)-N(2A)-C(3A)-N(8A)	−177.1(6)
N(1A)-N(2A)-C(3A)-N(4A)	1.0(7)
C(9A)-N(8A)-C(3A)-N(2A)	−11.5(10)
C(9A)-N(8A)-C(3A)-N(4A)	170.6(6)
C(5A)-N(4A)-C(3A)-N(2A)	−1.0(7)
C(5A)-N(4A)-C(3A)-N(8A)	177.2(6)
N(2A)-N(1A)-C(5A)-N(4A)	0.1(7)
C(6A)-N(1A)-C(5A)-N(4A)	−177.3(6)
C(3A)-N(4A)-C(5A)-N(1A)	0.5(7)

C(5A)-N(1A)-C(6A)-C(7A)	−113.2(8)
N(2A)-N(1A)-C(6A)-C(7A)	69.5(8)
C(3A)-N(8A)-C(9A)-P(14A)	−88.7(6)
C(3A)-N(8A)-C(9A)-P(10A)	143.5(5)
O(16A)-P(14A)-C(9A)-N(8A)	−176.6(4)
O(15A)-P(14A)-C(9A)-N(8A)	58.3(5)
O(17A)-P(14A)-C(9A)-N(8A)	−53.7(5)
Ca(1)-P(14A)-C(9A)-N(8A)	−155.2(4)
O(16A)-P(14A)-C(9A)-P(10A)	−54.9(4)
O(15A)-P(14A)-C(9A)-P(10A)	−180.0(3)
O(17A)-P(14A)-C(9A)-P(10A)	68.1(4)
Ca(1)-P(14A)-C(9A)-P(10A)	−33.4(4)
O(11A)-P(10A)-C(9A)-N(8A)	29.1(5)
O(13A)-P(10A)-C(9A)-N(8A)	156.7(4)
O(12A)-P(10A)-C(9A)-N(8A)	−85.3(5)
Ca(1)#1-P(10A)-C(9A)-N(8A)	51.8(4)
O(11A)-P(10A)-C(9A)-P(14A)	−93.7(4)
O(13A)-P(10A)-C(9A)-P(14A)	34.0(5)
O(12A)-P(10A)-C(9A)-P(14A)	152.0(4)
Ca(1)#1-P(10A)-C(9A)-P(14A)	−70.9(4)
N(1B)-N(2B)-C(3B)-N(8B)	−178.8(6)
N(1B)-N(2B)-C(3B)-N(4B)	0.9(7)
C(9B)-N(8B)-C(3B)-N(2B)	−6.8(10)
C(9B)-N(8B)-C(3B)-N(4B)	173.5(6)
C(5B)-N(4B)-C(3B)-N(2B)	−1.3(7)
C(5B)-N(4B)-C(3B)-N(8B)	178.5(6)
N(2B)-N(1B)-C(5B)-N(4B)	−0.5(7)
C(6B)-N(1B)-C(5B)-N(4B)	179.7(6)
C(3B)-N(4B)-C(5B)-N(1B)	1.0(7)
C(5B)-N(1B)-C(6B)-C(7B)	−115.6(8)
N(2B)-N(1B)-C(6B)-C(7B)	64.6(8)
C(3B)-N(8B)-C(9B)-P(10B)	128.0(5)
C(3B)-N(8B)-C(9B)-P(14B)	−105.4(6)
O(12B)-P(10B)-C(9B)-N(8B)	−169.4(4)
O(11B)-P(10B)-C(9B)-N(8B)	−43.7(6)
O(13B)-P(10B)-C(9B)-N(8B)	66.6(5)
Ca(1)-P(10B)-C(9B)-N(8B)	171.8(4)
O(12B)-P(10B)-C(9B)-P(14B)	67.6(4)
O(11B)-P(10B)-C(9B)-P(14B)	−166.8(4)
O(13B)-P(10B)-C(9B)-P(14B)	−56.5(5)
Ca(1)-P(10B)-C(9B)-P(14B)	48.7(3)
O(17B)-P(14B)-C(9B)-N(8B)	−46.7(5)
O(16B)-P(14B)-C(9B)-N(8B)	−172.3(4)
O(15B)-P(14B)-C(9B)-N(8B)	72.1(5)
O(17B)-P(14B)-C(9B)-P(10B)	78.0(4)
O(16B)-P(14B)-C(9B)-P(10B)	−47.6(5)
O(15B)-P(14B)-C(9B)-P(10B)	−163.2(4)

Table S7. Hydrogen bonds for 121_a (compound **2**) [Å]. Symmetry transformations used to generate equivalent atoms: #1 −x + 2, −y + 1, −z + 1; #2 −x + 3, −y + 1, −z + 1; #3 −x + 1, −y, −z + 1.

D-H…A	D(D-H)	D(H…A)	D(D…A)	<(DHA)
N(8A)-H(8AA)…O(11A)	0.86	2.39	2.822(7)	111.3
O(17A)-H(17A)…O(13A) #1	0.84	1.84	2.613(6)	151.8
O(20)-H(20A)…O(21) #4	0.839(10)	2.25(17)	2.80(4)	124(16)
O(20)-H(20A)…O(22) #4	0.839(10)	1.89(14)	2.575(18)	138(19)
O(20)-H(20B)…O(20) #4	0.840(10)	1.78(15)	2.40(3)	129(18)
O(20)-H(20B)…O(21)	0.840(10)	1.71(18)	1.94(4)	92(13)
O(20)-H(20B)…O(22)	0.840(10)	2.5(2)	2.88(2)	106(16)
O(21)-H(21D)…O(20)	0.840(10)	1.23(18)	1.94(4)	137(29)
O(22)-H(22A)…N(2A) #5	0.841(10)	2.25(8)	2.818(10)	125(8)
O(22)-H(22B)…O(20) #4	0.844(10)	2.02(8)	2.575(18)	123(8)
O(23)-H(23A)…O(16A) #5	0.840(10)	2.25(16)	2.768(13)	120(16)
C(5A)-H(5AB)…O(12A) #6	0.93	2.39	3.280(9)	160
